# Supplementary material for: Feeding ecology and activity patterns of Hippopotamus amphibious in the Dhidhessa Wildlife Sanctuary, Southwestern Ethiopia
Source: PLoS One. 2025 Aug 8;20(8):e0325731. doi: 10.1371/journal.pone.0325731 (PMC12334000; doi:10.1371/journal.pone.0325731)
Supplement: S1 Appendix — (DOCX) [file pone.0325731.s001.docx]

**Supplementary**

S1Table: Data sheet used for behavioral activity and event patterns (daily cycle)

| Time of the day | Activity | | | | | | |
| --- | --- | --- | --- | --- | --- | --- | --- |
|  | Feeding | Walking | Resting | Standing | Socializing | Barking | Yawing |
| 6:00-7:00 |  |  |  |  |  |  |  |
| 7:00-8:00 |  |  |  |  |  |  |  |
| 8:00-9:00 |  |  |  |  |  |  |  |
| 9:00-10:00 |  |  |  |  |  |  |  |
| 10:00-11:00 |  |  |  |  |  |  |  |
| 11:00-12:00 |  |  |  |  |  |  |  |
| 12:00-13:00 |  |  |  |  |  |  |  |
| 13:00-14:00 |  |  |  |  |  |  |  |
| 14:00-15:00 |  |  |  |  |  |  |  |
| 15:00-16:00 |  |  |  |  |  |  |  |
| 16:00-17:00 |  |  |  |  |  |  |  |
| 17:00-18:00 |  |  |  |  |  |  |  |

S2 Table: Data sheet used for behavioral activity and event patterns by adult hippopotamus (monthly)

| Time of the day | Feeding | | Walking | | Resting | | Standing | | Socializing | | Barking | | Yawing | |
| --- | --- | --- | --- | --- | --- | --- | --- | --- | --- | --- | --- | --- | --- | --- |
|  | M | F | M | F | M | F | M | F | M | F | M | F | M | F |
| Jan |  |  |  |  |  |  |  |  |  |  |  |  |  |  |
| Feb |  |  |  |  |  |  |  |  |  |  |  |  |  |  |
| Mar |  |  |  |  |  |  |  |  |  |  |  |  |  |  |
| Apr |  |  |  |  |  |  |  |  |  |  |  |  |  |  |
| May |  |  |  |  |  |  |  |  |  |  |  |  |  |  |
| Jun |  |  |  |  |  |  |  |  |  |  |  |  |  |  |

Key; - M= Male, F= Female

S3 Table: Mean percentage of food items identified from faecal analysis of hippopotamus in the study area

| Wet season | | | | | Dry season | | | |
| --- | --- | --- | --- | --- | --- | --- | --- | --- |
| Samples | Graminoids | Herbs | Dicots | Unknown | Graminoids | Herbs | Dicots | Unknown |
| 1 | 50 | 30 | 10 | 10 | 50 | 20 | 20 | 10 |
| 2 | 60 | 20 | 20 | - | 80 | 20 | - | - |
| 3 | 40 | 50 | 10 | - | 50 | 20 | 20 | 10 |
| 4 | 30 | 40 | 10 | 20 | 90 | 0 | 10 | - |
| 5 | 50 | 30 | - | 20 | 60 | 0 | 30 | 10 |
| 6 | 70 | 20 | - | 10 | 70 | 20 | - | 10 |
| 7 | 70 | 30 | - | - | 80 | 0 | 20 | - |
| 8 | 40 | 40 | 20 | - | 50 | 30 | - | 20 |
| 9 | 60 | - | 30 | 10 | 60 | 10 | 10 | 20 |
| 10 | 50 | 30 | 20 | - | 80 | 10 | 10 | - |
| 11 | 60 | 30 | - | 10 | 90 | 0 | - | 10 |
| 12 | 40 | 50 | 10 | - | 70 | 20 | 10 | - |
| 13 | 70 | 30 | - | - | 30 | 30 | 30 | 10 |
| 14 | 80 | 20 | - | - | 70 | 30 | - | - |
| 15 | 50 | 30 | 20 | - | 40 | 30 | 20 | 10 |
| 16 | 80 | 20 | - | - | 60 | 0 | 30 | 10 |
| 17 | 60 | - | 30 | 10 | 50 | 30 | 20 | - |
| 18 | 50 | 40 | - | 10 | 60 | 40 | - | - |
| 19 | 30 | 60 | 10 | - | 70 | 0 | 20 | 10 |
| 20 | 50 | 20 | 20 | 10 | 80 | 20 | - | - |
| Mean | 54.5 | 29.5 | 10.5 | 5.5 | 64.5 | 16.5 | 12.5 | 6.5 |


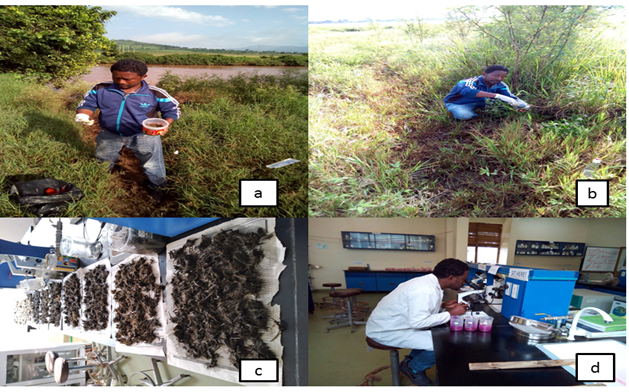


Figure S1: Images illustrating faecal analysis in microscopes at the laboratory of biology department of Wollega University (a-b) = Sample taken of faecal droppings of hippopotamus, (c) = air-dried and (d) = identification processes of specimens.


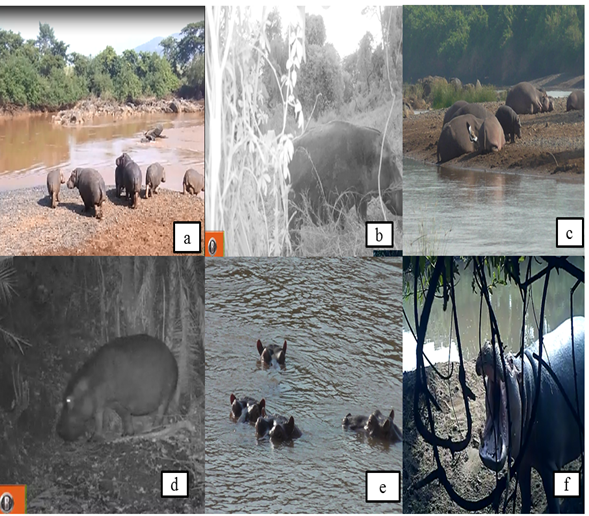


Figure S2: Distribution and different behavioral activities of hippo in the DWS (a-e) = activities and (f) event


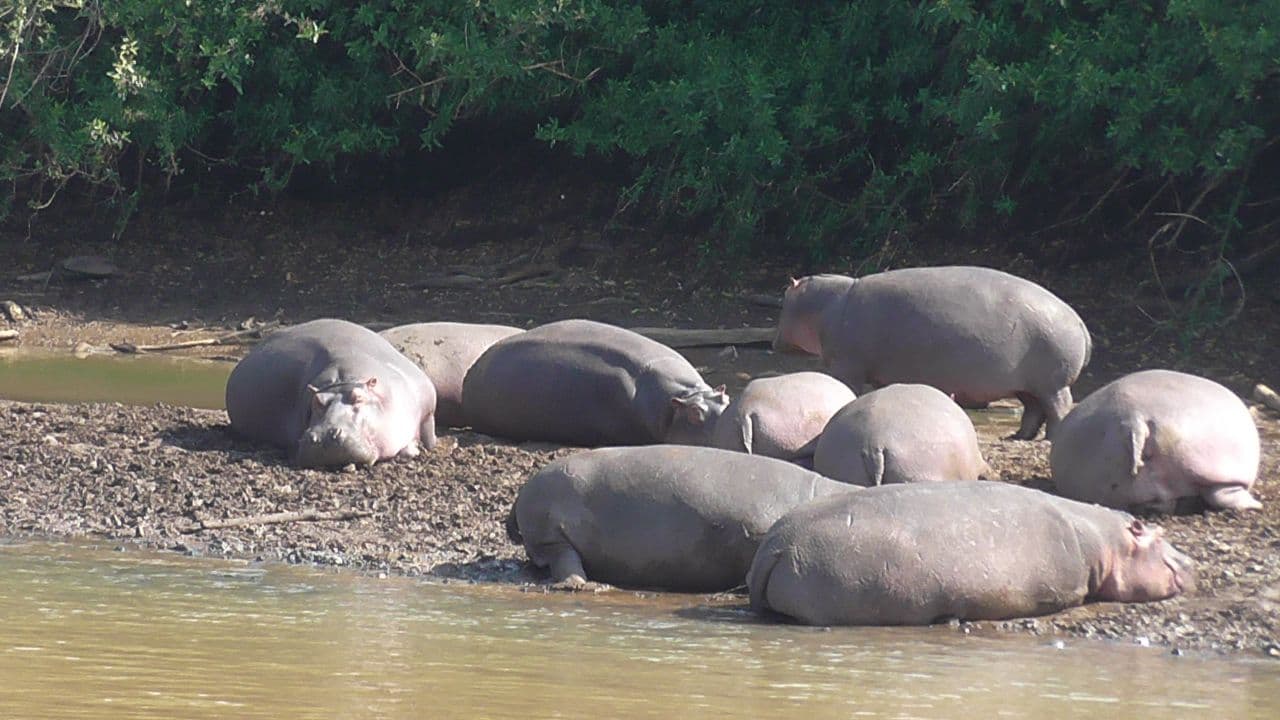


Figure S3: Resting and standing activities of the Common Hippopotamus in the DWS


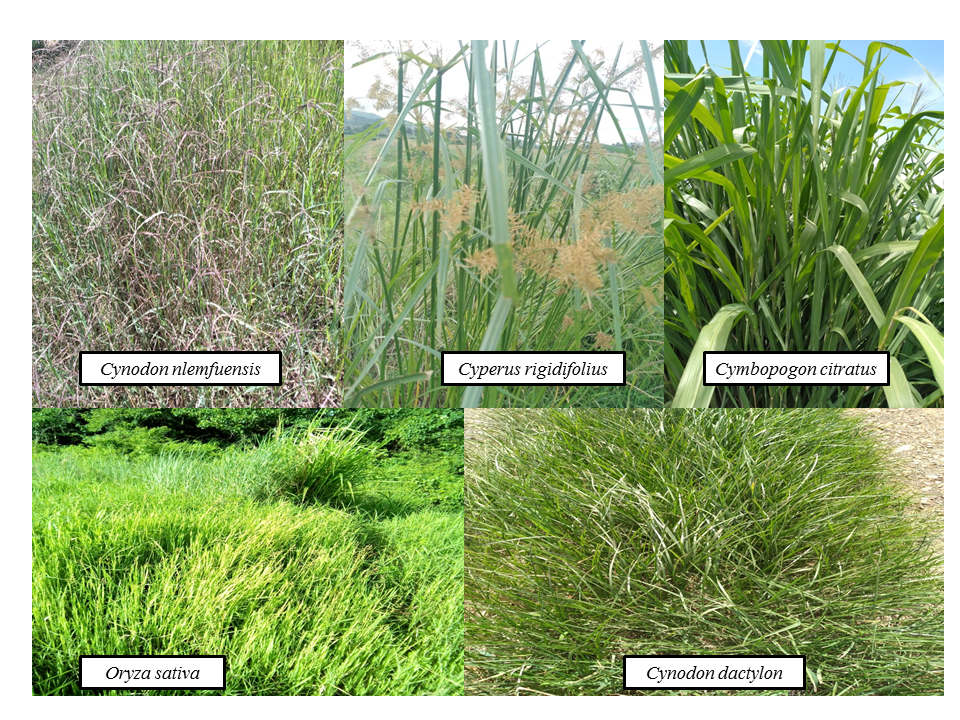


Figure S4: Some of the grass species foraged by hippopotamus in DWS
