# Supplementary material for: Feeding ecology and activity patterns of Hippopotamus amphibious in the Dhidhessa Wildlife Sanctuary, Southwestern Ethiopia
Source: PLoS One. 2025 Aug 8;20(8):e0325731. doi: 10.1371/journal.pone.0325731 (PMC12334000; doi:10.1371/journal.pone.0325731)
Supplement: Attachment — Submitted filename: PONE-D-24-55622_reviewer Hippopaper.pdf [file pone.0325731.s002.pdf]

PLOS ONE

Feeding ecology and activity patterns of Common Hippopotamus in the Dhidhessa Wildlife Sanctuary, Southwestern Ethiopia

--Manuscript Draft--

**Manuscript Number:**	PONE-D-24-55622
**Article Type:**	Research Article
**Full Title:**	Feeding ecology and activity patterns of Common Hippopotamus in the Dhidhessa Wildlife Sanctuary, Southwestern Ethiopia
**Short Title:**	Ecology of Common Hippopotamus in the Dhidhessa Wildlife Sanctuary, Southwestern Ethiopia
**Corresponding Author:**	Girma Gizachew Tefera, MSC<br>Jimma University College of Agriculture and Veterinary Medicine<br>Jimma, ETHIOPIA
**Keywords:**	Behavioral activity, Common Hippopotamus, Dhidhessa Wildlife Sanctuary, Feeding ecology.
**Abstract:**	The study of activity patterns and dietary composition for hippos is essential for understanding the links between this species and the environment in which it lives. The objective of this research was to examine the activity patterns and dietary habits of common hippopotamuses in the Dhidhessa Wildlife Sanctuary (DWS) southwest, Ethiopia. The survey was carried out between 2022 and 2023. Event patterns and behavioral activity were studied with 30-minute intervals following a male and female as focal sampled individuals. In order to study the hippopotamus' diet, plant species identified from fresh eating signals were gathered, and supplemented by fecal analysis. With 30.1% of their time spent resting, they made up the biggest amount of all behavioral activities, followed by moving (23.7%). 53.1% of their time was spent barking, while 46.9% was spent yawning. Compared to females, males rested more. With a resting peak in the middle of the day, feeding and movement peak activities were noted in the early morning and late afternoon. Both male and female hippopotamuses exhibited a greater frequency of barking and yawning in the afternoon. Thirty-four plant species from twelve families were devoured by hippos. Family Poaceae contributed the largest (60%), proportion of their diet while Combretaceae is the least (0.5%). Based on the overall percentage contribution, Typha latifolia was the most consumed plant species, which accounted for 9.4% followed by Eriochloa fatmensis (8.7%) while, Oryza sativa and Chamaeurista mimosoides (0.3%, for each). Therefore, DWS's primary priority should be creating management plans to reestablish the sanctuary as a fully functional sustainable ecosystem.
**Order of Authors:**	Girma Gizachew Tefera, MSC
	Tadesse Habtamu Tessema, Dr
	Tibebu Alemu Bekere, Dr
	Tariku Mekonnen Gutema, Dr
**Additional Information:**	
**Question**	**Response**
**Financial Disclosure**<br><br>Enter a financial disclosure statement that describes the sources of funding for the work included in this submission. Review the submission guidelines for detailed requirements. View published research articles from *PLOS ONE* for specific	The author(s) received no specific funding for this work.

Powered by Editorial Manager® and ProduXion Manager® from Aries Systems Corporation

examples.<br><br>This statement is required for submission and **will appear in the published article** if the submission is accepted. Please make sure it is accurate.<br><br>**Funded studies**<br>Enter a statement with the following details:<br>• Initials of the authors who received each award<br>• Grant numbers awarded to each author<br>• The full name of each funder<br>• URL of each funder website<br>• Did the sponsors or funders play any role in the study design, data collection and analysis, decision to publish, or preparation of the manuscript?<br><br>Did you receive funding for this work?	
**Competing Interests**<br><br>Use the instructions below to enter a competing interest statement for this submission. On behalf of all authors, disclose any competing interests that could be perceived to bias this work—acknowledging all financial support and any other relevant financial or non-financial competing interests.<br><br>This statement is required for submission and **will appear in the published article** if the submission is accepted. Please make sure it is accurate and that any funding sources listed in your Funding Information later in the submission form are also declared in your Financial Disclosure statement.<br><br>View published research articles from *PLOS ONE* for specific examples.	The authors declare no competing interests

**NO authors have competing interests**<br><br>Enter: *The authors have declared that no competing interests exist.*<br><br>**Authors with competing interests**<br><br>Enter competing interest details beginning with this statement:<br><br>*I have read the journal's policy and the authors of this manuscript have the following competing interests: [insert competing interests here]*<br><br>* typeset	
**Ethics Statement**<br><br>Enter an ethics statement for this submission. This statement is required if the study involved:<br><br>• Human participants<br>• Human specimens or tissue<br>• Vertebrate animals or cephalopods<br>• Vertebrate embryos or tissues<br>• Field research<br><br>Write "N/A" if the submission does not require an ethics statement.<br><br>General guidance is provided below. Consult the submission guidelines for detailed instructions. **Make sure that all information entered here is included in the Methods section of the manuscript.**	Ethical approval<br>The research proposal (supplementary file) was reviewed and granted approval by the Review Board at Jimma University (JU) College of Agriculture, Veterinary, and Medicine. The vice president's office also reauthorized and accepted the request for research and community service from the review board. JU sanctioned the process for collecting consent needed for the data collection tools (RGS/752/2021). Consent to participation is not applicable since no respondents were participated in this research work. All methods used in this study were performed in accordance with the relevant guidelines and regulations.

Powered by Editorial Manager® and ProduXion Manager® from Aries Systems Corporation

**Format for specific study types**<br><br>**Human Subject Research (involving human participants and/or tissue)**<br>• Give the name of the institutional review board or ethics committee that approved the study<br>• Include the approval number and/or a statement indicating approval of this research<br>• Indicate the form of consent obtained (written/oral) or the reason that consent was not obtained (e.g. the data were analyzed anonymously)<br><br>**Animal Research (involving vertebrate animals, embryos or tissues)**<br>• Provide the name of the Institutional Animal Care and Use Committee (IACUC) or other relevant ethics board that reviewed the study protocol, and indicate whether they approved this research or granted a formal waiver of ethical approval<br>• Include an approval number if one was obtained<br>• If the study involved *non-human primates*, add *additional details* about animal welfare and steps taken to ameliorate suffering<br>• If anesthesia, euthanasia, or any kind of animal sacrifice is part of the study, include briefly which substances and/or methods were applied<br><br>**Field Research**<br><br>Include the following details if this study involves the collection of plant, animal, or other materials from a natural setting:<br>• Field permit number<br>• Name of the institution or relevant body that granted permission	
**Data Availability**<br><br>Authors are required to make all data underlying the findings described fully available, without restriction, and from the time of publication. PLOS allows rare exceptions to address legal and ethical concerns. See the PLOS Data Policy and FAQ for detailed information.	Yes - all data are fully available without restriction

Powered by Editorial Manager® and ProduXion Manager® from Aries Systems Corporation

A Data Availability Statement describing where the data can be found is required at submission. Your answers to this question constitute the Data Availability Statement and **will be published in the article**, if accepted.<br><br>**Important:** Stating 'data available on request from the author' is not sufficient. If your data are only available upon request, select 'No' for the first question and explain your exceptional situation in the text box.<br><br>Do the authors confirm that all data underlying the findings described in their manuscript are fully available without restriction?	
**Describe where the data may be found in full sentences. If you are copying our sample text, replace any instances of XXX with the appropriate details.**<br><br>• If the data are **held or will be held in a public repository**, include URLs, accession numbers or DOIs. If this information will only be available after acceptance, indicate this by ticking the box below. For example: *All XXX files are available from the XXX database (accession number(s) XXX, XXX.*).<br>• If the data are all contained **within the manuscript and/or Supporting Information files**, enter the following: *All relevant data are within the manuscript and its Supporting Information files.*<br>• If neither of these applies but you are able to provide **details of access elsewhere**, with or without limitations, please do so. For example:<br><br>*Data cannot be shared publicly because of [XXX]. Data are available from the XXX Institutional Data Access / Ethics Committee (contact via XXX) for researchers who meet the criteria for access to confidential data.*<br><br>*The data underlying the results presented in the study are available from (include the name of the third party*	All relevant data is within the paper and it's supporting information files

and contact information or URL).<br>• This text is appropriate if the data are owned by a third party and authors do not have permission to share the data.<br><br>* typeset	
Additional data availability information:	Tick here if the URLs/accession numbers/DOIs will be available only after acceptance of the manuscript for publication so that we can ensure their inclusion before publication.

Powered by Editorial Manager® and ProduXion Manager® from Aries Systems Corporation

Manuscript Click here to access/download;Manuscript;Manuscript.docx

Feeding ecology and activity patterns of Common Hippopotamus in the Dhidhessa Wildlife Sanctuary, Southwestern Ethiopia

Girma Gizachew Tefera[1], Tadesse Habtamu Tessema[2] , Tibebu Alemu Bekere[3], Tariku Mekonnen Gutema[4]

[1] Department of Natural Resource Management, Colleague of Agriculture and Veterinary Medicine, Jimma University, Jimma, Ethiopia.

[2] Department of Biology, College of computational Sciences, Jimma University, Jimma, Ethiopia

[3] Department of Natural Resource Management, Colleague of Agriculture and Veterinary Medicine, Jimma University, Jimma, Ethiopia.

[4] Department of Natural Resource Management, Colleague of Agriculture and Veterinary Medicine, Jimma University, Jimma, Ethiopia.

Corresponding author: kennaa20047@gmail.com
Tel: +251917550062

Abstract

The study of activity patterns and dietary composition for ~~hippos~~ is essential for understanding the links between this species and the environment in which it lives. The objective of this research was to examine the activity patterns and dietary habits of common hippopotamuses in the Dhidhessa Wildlife Sanctuary (DWS) southwest, Ethiopia. The survey was carried out between 2022 and 2023. Event patterns and behavioral activity were studied with 30-minute intervals following a ~~male and female~~ as focal sampled individuals. In order to study the hippopotamus' diet, plant species identified from ~~fresh eating signals~~ were gathered, ~~and supplemented by fecal analysis~~. ~~With 30.1% of their time spent resting, they made up the biggest amount of all behavioral activities, followed by moving (23.7%).~~ 53.1% of their time was spent barking, while 46.9% was spent yawning. ~~Compared to females, males rested more~~. With a resting peak in the middle of the day, feeding and movement peak activities were noted in the early morning and late afternoon. Both male and female hippopotamuses exhibited a greater frequency of ~~barking~~ and yawning in the afternoon. Thirty-four plant species from twelve families were devoured by hippos. Family Poaceae contributed the largest (60%), proportion of their diet while Combretaceae is the least (0.5%). Based on the overall percentage contribution, Typha latifolia was the most consumed plant species, which accounted for 9.4% followed by Eriochloa fatmensis (8.7%) while, Oryza sativa and Chamaeurista mimosoides (0.3%, for each). Therefore, ~~DWS's~~ primary priority should be creating management plans to reestablish the sanctuary as a fully functional sustainable ecosystem.

Keywords: Behavioural activity, Common Hippopotamus, Dhidhessa Wildlife Sanctuary, Feeding ecology.

Introduction

Background of the study

The common hippopotamus, *Hippopotamus amphibius L*, (hereafter referred to as hippo), was once widespread across wet habitats throughout Africa [1], particularly in lakes and rivers of sub-Saharan Africa [2] . In terms of size and weight, it ranks third among terrestrial mammals, behind white rhinos and elephants [3] . The recognized species of hippo is distinguished by its large, round head, short bulky limbs, broad nose, short tail, short stocky legs, and very long tusk-like canines. They have eyes, ears, and nostrils on the high roof of the cranium [4] . Due to their thin epidermis, hippos are prone to desiccation. The natural sunscreen chemical "blood sweat" that their skin secretes helps shield them from the sun. Despite this, because they lack sweat glands, their skin will fracture if exposed to air for too long[5] .

Hippos are considered obligatory herbivores, meaning they favor plants and short grass [6,7]. Animal evolution, life-history techniques, and ecological role are all shaped by their diets [8] . Food is one of the niche's most important dimensions. As a result, most ecological studies require knowledge of animal diets. The study of animal diets is essential for understanding the links between species and the environment in which they live [9,10]. These relationships may determine community structure, species diversity, relative abundance, and resource partitioning among species and individuals.

As the name implies, the *Hippopotamus amphibius* is an amphibious animal with two primary habitat needs: grass for nighttime grazing and water for daytime repose [11]. Hippos can be found in a variety of permanent freshwater environment, including ponds, rivers, lakes, and waterholes with gently sloping banks surrounded by grazing areas. It avoids fast-moving streams

with heavily swampy vegetation and rocky outcroppings [1]. The hippos are a selective grazer that consumes 35–50 kg of grass per day, or roughly 2.5% of its body weight [12, 13]. Unlike other mega-herbivores, it needs both an open grazing range and a place to live in water on a daily basis [5].

The research on the ecology and behavior of hippos, as well as the invasive and often antagonistic relationship between humans and hippos, is still lacking [14]. These points have led to the formulation of the objective of this study. The intended purposes for which the DWS, Southwestern Ethiopia, was formed have not been met recently. Large mammal populations in the vicinity subsequently moved into the adjacent forest patches. For one thing, the DWS is home to a variety of large mammals, including hippopotamuses. Hence, the current study area focused on the feeding ecology and behavioral activity of the common hippo in order to develop long-term conservation measures in the study area, all of which will help to establish a national park in the area.

Materials and methods

Ethical statement

The research proposal (supplementary file) was reviewed and granted approval by the Review Board at Jimma University (JU) College of Agriculture, Veterinary, and Medicine. The vice president's office also reauthorized and accepted the request for research and community service from the review board. JU sanctioned the process for collecting consent needed for the data collection tools (RGS/752/2021). Consent to participation is not applicable since no respondents were participated in this research work. All methods used in this study were performed in accordance with the relevant guidelines and regulations.

Description of the Study Area

The study was conducted in the DWS and its surrounding areas in southwestern Ethiopia, spanning three administrative zones: Jimma, Buno Bedele, and East Wollega, all within the Oromia National Regional State. The total area is approximately 1300 km². It is situated 395 km West of Addis Ababa, along the Addis Ababa Nekemte-Bedele route. Geographically, the location is between $8^0$30’0” and $8^0$40’0” N latitude and $36^0$22’0” and $36^0$43’ 0” E longitude (Fig.1 our team used shape files provided by the Ethiopian Mapping Agency to produce the map. The agency’s official website offers free access to these shape files (http://www.ema.gov.et). The altitude in the region varies from 1050 to 2560 meters. The area receives annual precipitation in a uni-modal pattern, ranging from 648 to 2001.8 mm. The Sanctuary has two distinct seasons: a dry season from November to February and a wet season from June to September. The lowland areas within the sanctuary tend to have warmer temperatures, with average minimum and maximum annual temperatures of 12°C and 35°C, respectively [15]. The predominant vegetation types in the study area include *Combretum*, *Commiphora*, and *Acacia* species, interspersed with a primarily growing savanna grass composed of *Hyparrhenia* species.

Methods

In order to record the activity patterns of hippos in the study area, a focal animal sampling method was used, focusing on two focal sampled animals were selected using the methodology suggested by [17] in this same area Data on the daytime behaviors of a pair of focal animals—an adult male and an adult female—were recorded every 30 minutes, with a 5-minute sampling interval in between. ~~Focal animals were selected randomly and clearly identified by distinctive morphological characteristics (skin mark or skin colour) by using binoculars or naked eyes.~~ Additionally, a group or individual hippos was rapidly scanned by using camera traps at the

sampling location, which were scanned at five-minute intervals every 24 hours during the dry (January to March) and rainy seasons (May to July), and the behavior of each animal was recorded at that instant. Observations were conducted for five consecutive days in each of the six months. A total of 120 days of behavioural observations were done in both seasons. During each season, behavioral activity was recorded as resting, standing, walking, feeding, and socializing, while yawning and barking were recorded as behavioral events every five minutes (Table S1 and S2). These behavioral categories, however, were referred to as behavioral actions and occurrences [17, 18]. [16] Assert that behavioral events are a shorter period of time, whereas behavioral activities are long-duration.

Based on information from a preliminary survey in the study area, the plant species that hippos eat were investigated by looking at new feeding indicators following focal groups at close distances [13]. The food plants the animal had bitten or plucked were collected, compressed, and identified with the help of a taxonomist from the Botanical Herbarium at Wollega University. To calculate a plant species' percentage of feeding preference, the frequency of biting or feeding sign observations for that species were added up, divided by the total frequency of all plant species gathered at feeding sites, and then multiplied by 100 over the course of the study, $CSi = \frac{FSi}{\sum_{i=1}^{n} FSt} x100$, where FSi is the frequency of species i, which corresponds to the number of hits of the species along transects within a plot CSi, the contribution of species i to the sum of the frequencies of all species; it reflects the abundance of a species and expressed as a percentage and $\sum_{i=1}^{n} \mathrm{FSt} =$ the total frequency of all collected species.

The fecal analysis method was used in conjunction with the feeding observation technique to identify species that were foraged [19-21]. Dung samples were gathered from various habitats and air-dried prior to analysis [22] (Figure S1). Fecal samples collected during the same season

were combined to create composite samples [23] These composite samples were manually broken apart and thoroughly washed with water using a 0.1 mm sieve to eliminate finely digested food particles [24] . Subsequently, samples were placed into a beaker, treated with 75% alcohol, and allowed to sit for 48 hours. After the alcohol was decanted, domestic bleach was introduced and left to react until the bleaching was complete [26, 26]. The samples were then rinsed with water, stained with safranin, and allowed to sit for 2 days [25] prior to being examined microscopically. Ten slides from each composite fecal sample were analyzed using a compound light microscope. A small portion of the prepared fecal sample was applied to a gridded slide and counted under ×100 and × 40 magnifications [27]. The counting and identification of epidermal fragments were supported by reference slides [28] and by examining morphological characteristics such as cell shape, venation, and trichomes [28]. Species-level identified fragments were categorized into graminoids, herbs, and dicots [22, 29], while those that could not be identified were classified as unknown.

Results

Diurnal activity and event patterns of hippopotamus

A total of 4,480 behavioral records were collected from adult male and female common hippos through focal sampling. Among these records, 43.3% (1,941) were categorized as activities, while the remaining 56.7% (2,539) were events documented during a total of 540 hours of behavioral observation. Furthermore, the observational data indicated that hippos spent a significantly greater proportion of their time in resting activities (30.1%) compared to other types of activities (see Table 1). However, ~~barking and yawning~~ events constituted 53.1% and 46.8% of the observed behaviors, respectively (Figure S2).

Time-specific activity budgets

Certain aspects of the activity budget for both male and female hippopotamuses exhibited notable variations throughout the day. The behaviors of feeding, moving, resting, and standing changed significantly at different times over the course of the study (Fig. 2). Feeding, moving, and standing behaviors each displayed two peaks: one in the early morning hours from 07:00 to 09:00 and another in the late afternoon from 16:00 to 18:00. The behaviors of resting or lying down were particularly prominent during the hottest parts of the day, specifically from 11:00 to 15:00. The final activity noted for both male and female hippopotamuses was socialization, which included mating behaviors. Instances of ~~barking and yawning~~ were recorded throughout the day in both sexes, with a marked increase in the afternoon. The yawning behavior involves the animal raising its head and extending its mouth forward (Fig. 3). After yawning, they tend to open and close their mouths repeatedly while emitting low grunts and engaging in more head bobbing. The yawning behaviors of male and female hippopotamuses were found to be quite similar.

Monthly variations in activity and event budget

The highest frequency of feeding occurred in June, followed by May, while January and February had the lowest feeding occurrences (Table 2). June also recorded the greatest amount of movement. The levels of movement in February and July were relatively similar. March had the highest resting figures, with February following, while January and July recorded the least moving and standing, respectively. The peak social interaction among hippos was noted in February and March, whereas July saw the lowest socializing levels. March had the most frequent ~~barking~~ events, with February in second place, while July had the fewest incidences of ~~barking~~. The most yawning among hippos was observed in March, followed by February, whereas July had the least yawning, closely followed by May (Table 2).

Comparison in activity and event budget

The distribution of time that adult females and males spent on various activities and events throughout the study period as displayed in Table 1. Among the 1941 and 2539 behavioral activities and events recorded, male hippopotamuses exhibited a lower percentage (48.7% and 47.6%) of these activities compared to females (51.3% and 52.4%). Females engage in more behavioral activities than their male counterparts.

During the daytime, male hippos devoted 32.2% of their activity to resting. The next most common activity was standing, which accounted for 19.8%, followed by feeding at 18.9% (refer to Figure S3). In terms of the daytime activities of female hippos, the highest recorded activities were moving (28.9%) and resting (28%), with feeding following closely at 24%. However, there were no significant differences observed in the activity patterns between male and female hippos ($t=2.18$, $df= 6$, $P>0.05$) (Fig.4).

Seasonal variations in activity budget

During the wet season, there were a total of 1110 recorded behavioral activities, while the dry season accounted for 831. Behavioral events were documented in both the rainy (1070) and dry (1469) seasons. In the dry season, hippopotamuses allocated their time to resting (37.1%), standing (17.8%), and barking (54.3%), with these activities being more pronounced compared to the wet season. Conversely, in the wet season, the hippos showed a higher frequency of feeding (28.9%), walking (31.8%), and yawning (48.5%) when compared to the dry season (Fig.5). Significant differences were observed in the activity budgets between the dry and wet seasons for hippos ($t=2.44$, $df =6$, $p<0.05$).

Feeding ecology

A total of 34 plant species from 12 families (excluding crops) were identified (Table 3). Among these, 23 species were documented through observations of feeding signs and daytime feeding records during focal sampling, while the other 11 species were noted based on observed feeding bites. The Poaceae family made the largest contribution to their diet, constituting 60%, whereas Combretaceae had the smallest share at 0.5% (Figure S4). In terms of overall percentage contribution, *Typha latifolia* emerged as the most consumed plant species, making up 9.4%, followed closely by *Eriochloa fatmensis* at 8.7%. Meanwhile, *Oryza sativa* and *Chamaeurista mimosoides* each represented 0.3%, with *Psoralea bituminosa* following at 0.2% (Table 3).

Alongside the species present in the DWS, crops such as Maize (*Zea mays*), Sugar cane (*Saccharum officinarum*), Banana (*Musa paradisiaca*), Great millet (*Sorghum bicolor*), Garlic (*Allium sativum*), and Onion (*Allium cepa*) were significantly consumed by the hippopotamus in agricultural areas.

During the wet season, hippos were observed eating a wider range of forage species (76.7%) in contrast to the dry season (23.3%). The most frequently eaten forage species by hippos in the rainy season included *Typha latifolia* (9.9%), *Eriochloa fatmensis* (9.2%), *Alternanthera nodiflora* (7.7%), *Cyperus rigidifolius* (7.4%), and *Cynodon dacylon* (6.9%). In contrast, the dry season's predominantly consumed plant species were *Echinochloa pyramidalis* (15.1%), *Typha latifolia* (7.9%), *Cynodon nlemfuensis* (7.5%), *Eriochloa fatmensis* (7.2%), along with *Echinochloa cruspavonis* and *Cyperus rigidifolius* (both at 6.8%). Nonetheless, during the wet season, the least frequently consumed forage species included *Vigna vexillata* and *Oryza sativa* (0.3% for each), and *Chamaeurista mimosoides* and *Psoralea bituminosa* (0.2% for each), while the dry season saw *Chamaeurista mimosoides* (0.4%) and *Hyparrhenia cymbaria*, *Coelorhachis*

afraurita, and *Vigna vexillata* (0.8% for each) as the lowest consumed forage species in the study area. Importantly, there were notable differences in the consumption of each plant species across the various seasons throughout the study period ($t = 2.03$, $df = 33$, $P < 0.05$) (Table 4).

An examination of 80 hippopotamus droppings showed that different quantities of dietary elements, such as dicots, grasses (graminoids), forbs (herbs), and unidentified substances, were ingested during both the wet and dry seasons (Table 5).

Discussion

Activity pattern

Examining the fundamental behaviors of hippopotamuses is essential for evaluating the well-being of hippo populations, their ecosystems, and the potential for lasting coexistence with local communities [6, 17]. The results of the present study carried out in DWS indicated that hippos participated in a range of activities during the day, primarily resting, along with walking, feeding, standing, and engaging in social interactions. This observation aligns with the results reported by [18] in Chebera Churchura National Park and [30] in Boye Wetland, Ethiopia. Conversely, the current study's results differ from [17], who noted that hippos rest for over half the day. This discrepancy may be attributed to the differing sampling methods, as this study utilized focal sampling while Timbuka employed scan sampling. Nevertheless, there were variations in behavioral events like ~~barking~~ and yawning among hippopotamuses.

The current study's findings revealed that the hippopotamus exhibits a general activity pattern marked by peaks in the morning and late afternoon, with a resting phase during midday. In the morning, they devoted several hours to feeding, frequently returning to the water, which made them somewhat agitated. During the daytime heat, most hippopotamuses were observed resting.

This behavior serves as a crucial adaptation for their survival and helps prevent their skin from drying out under the sun [31]. Around midday, the hippopotamus typically refrained from feeding, except on overcast or rainy days. A similar observation was made by [17, 18, 30], who noted that hippopotamuses often move and enter the water frequently during hot weather. The results of this study revealed that hippopotamuses did not entirely leave the water. This observation corresponds with studies carried out by [6,18,30,32, which reported that most of their bodies remained submerged while they extended their necks to graze on grasses floating on the surface or growing at the water's edge.

The current study's findings revealed that the hippopotamus exhibits a general activity pattern marked by peaks in the morning and late afternoon, with a resting phase during midday. In the morning, they devoted several hours to feeding, frequently returning to the water, which made them somewhat agitated. During the daytime heat, most hippopotamuses were observed resting. This behavior serves as a crucial adaptation for their survival and helps prevent their skin from drying out under the sun [31]. Around midday, the hippopotamus typically refrained from feeding, except on overcast or rainy days. A similar observation was made by [17, 18, 30], who noted that hippopotamuses often move and enter the water frequently during hot weather. The findings of the current study indicated that hippopotamuses did not completely exit the water. This finding aligns with research conducted by [6,18, 30,32], who noted that, most part of their body submersed in the water and extends the neck to the grasses that fluted on the water or grow at the edge parts have to forage.

The current study in DWS indicated that the activity budget percentage of hippopotamuses displayed variations throughout the months. This could be linked to factors such as the availability of a consistent water source, favored plant species near their resting areas, and

environmental temperatures, which significantly influenced the hippopotamus's activity patterns. Feeding activity was most prevalent in June, followed by May, and while it was least observed in February and March. During February and March, the temperatures rise and rainfall and cloud cover are minimal. As a result, hippopotamuses tended to rest during the day, often fully submerged in water or moving to shaded spots, and generally had less need to feed. Conversely, in June, the weather became cloudier, prompting increased activity as they searched for food and moved around. Feeding activity during the day also diminished in July, possibly because they had secured enough tender grass at night and faced reduced competition from livestock, leading to a decline in both feeding and resting activities during that month. The results of this research correspond with the outcomes of [18,30,33], who noted that increased water levels and flooding affect the animals' movements in search of food, causing the grass to submerge in water. Nonetheless, a notable increase in ~~barking~~ and yawning was recorded in March. Comparable results were observed by [6,18, 30], who observed that during March and April, hippopotamuses exhibited the highest frequency of yawning and ~~barking~~, likely related to mating behavior.

Research indicates that the behavioral differences between male and female hippopotamuses may be influenced by various factors, including disturbance, hunger, play, aggression, and mating activities. The results of the current study affirm that females engage in more behavioral activities than males. This result aligns with the observations made by [6], who noted that females exhibited greater aggression and vocalization when defending their young from threats posed by males or other disturbances. The female hippo fiercely guards her calf and displays behaviors such as ~~barking~~ and yawning. Conversely, both male and female hippos exhibit ~~barking~~ and yawning behaviors at any time throughout the day, with a notable increase during the afternoon. Similar observations were documented by [12, 18, and 30]. The seasonal patterns

of certain species may affect their behavioral variations across different seasons. The findings of the current study support the notion that during the dry season, hippos primarily engage in resting and standing, while the rainy season is associated with heightened rates of feeding and movement. Furthermore, due to competition and mating periods, barking and yawning behaviors were particularly prominent during the dry months. Similar observations were reported by [17, 34].

Feeding ecology

Research into the feeding habits of hippos is quite sparse, with many previous studies primarily focusing on qualitative data rather than quantitative analysis [35]. A diet survey of hippos conducted in the DWS and surrounding areas during this study found that they consumed 34 different plant species in both wet and dry seasons. This finding aligns with the research by [13] conducted in the Biosphere Reserve of Burkina Faso, which also identified 34 plant species, and [18] in Chebra Churchura National Park, Ethiopia, which noted 40 species. The higher number of species recorded in the study from Chebra Churchura National Park may be attributed to its well-preserved biodiversity with minimal human disruption, allowing for a wider range of plant species to be accessible to hippos. Conversely, the current study identified more plant species consumed by hippos compared to the research done by [36] in the Arjo Dhidhessa sugar factory, Ethiopia; [30] in the Boye Wetland, Ethiopia; and the Luangwa River in Zambia [12]. The disparity in the quantity of plant species eaten by hippos in this study area compared to earlier research may be influenced by the duration of the study periods and the decline in species diversity caused by human activities such as settlement expansion and agriculture. This finding is consistent with earlier studies conducted by [13] and [37]. In this study, various plant species were consumed by hippos, with *T. latifolia*, *E. fatmensis*, *C. rigidifolius*, *C. dactylon*, and

Sacciolepis africana being the most favored and constituting the largest part of their diet. Additionally, *C. dactylon* has frequently been identified as a primary food source for hippos in various nations. A comparable finding has been recorded by[12,18,36] .

The findings of this research revealed that almost all plant species were eaten by hippos during both seasons, except for *Alternanthera nodiflora*, *Orthosiphon schimperi*, *P. attenuato*, *Persicaria decipiens*, *Hyparrhenia glabriuscula*, *Panicum maximum*, *Oryza sativa*, *Psoralea bituminosa*, *Laburum anagyroide*, *Alternanthera nodiflora*, *Guizotia scabra*, and *Galinsoga parviflora*, which were not present in the dry season. Nevertheless, this suggests that a greater variety of species was consumed in the wet season compared to the dry season. As the dry season advanced, the diversity of species eaten by hippos declined. This alteration in the hippo's dietary composition may be attributed to the seasonal availability of various grass and herbaceous species in the study area. This observation aligns with the findings of [18, 36]. The types of forage eaten by hippos were divided into three groups: grasses, herbs, and shrubs. Of these, grasses made up the most significant part of the hippo's diet, comprising 66% in the study region. Numerous studies conducted in other locations have indicated that the feeding habits of hippos are predominantly comprised of grasses [5,6]. In the current study, the primary grasses from the Poaceae and Cyperceae families were significantly grazed during both the dry and wet seasons. The outcomes of the current research are consistent with the results of earlier studies conducted by [18, 36].

Conclusion

According to the results of this study, hippos have effectively adapted to thrive in DWS and the surrounding areas of southwestern Ethiopia. Among the various behavioral activities observed,

resting constituted the largest share for both males and females. Females displayed greater activity compared to males in movements, barking, and yawning. In contrast, males dedicated more time to resting than their female counterparts. Apart from social interactions, females were overall more active than males regarding their behaviors and activity patterns. Hippo activity levels peaked during the morning and late afternoon, while they tended to rest during the midday hours. There were variations in activity components and event patterns across different months and seasons. The dietary preferences of hippos reflect their ability to adapt to their surroundings, as they feed on a variety of plant species such as grasses, shrubs, and herbs. Habitats rich in vegetation and water are particularly favorable for hippos and other animals in the region. Although the forest environment remains largely intact and closed, the increasing human population, along with pressures from large herbivores, illegal settlements, agricultural expansion, and conflicts between humans and hippos (and other wildlife) could lead to excessive exploitation of limited resources in the area [38]. DWS has significant potential to support a considerable number of hippos and other wildlife, which could be developed into an ecotourism hub [39]. Therefore, it is strongly suggested that the Ethiopian Wildlife Authority take steps to reestablished the sanctuary designation of DWS and consider elevating it to national park status, while implementing necessary conservation measures, such as community-based conservation initiatives, to address existing issues. A thorough assessment of biodiversity in the area should be conducted to establish an effective long-term conservation and management strategy for hippos and other wildlife present.

Supporting information

Table S1: Data sheet used for behavioural activity and event patterns (daily cycle)

Table S2: Data sheet used for behavioural activity and event patterns by adult hippo (monthly).

Figure S1: Images illustrating faecal analysis in microscopes at the laboratory of biology department of Wollega University.

Figure S2: Distribution and different behavioural activities of hippo in the DWS.

Figure S3: Some of the grass species foraged by hippopotamus in DWS.

Acknowledgements: We thank to IDEA-WILD for their material support. The authors would like to acknowledge Arjo Dhidhessa Sugar Factory, local communities. And district experts who assisted the field survey. Also, they would like to thank Ashetu Kejela for preparing the map of the study area and Mokonnen Teshome for Botanical Herbarium, Wollega University and for diet quality analysis.

Authors' contributions: The study data collection was conceptualized and designed by Girma Gizachew, Tadesse Habtamu, Tariku Mekonnen, Tibebu Alemu. Girma Gizachew did the fieldwork, analyzed it, wrote the manuscript, and revised it all. Tadesse Habtamu, Tariku Mekonnen and Tibebu Alemu edited the manuscript and amended the primary document's final version before submitting it for evaluation. The authors participated in the manuscript's writing and approved the final version.

Data Availability: All relevant data is within the paper and it's supporting information files.

Funding: The author(s) received no specific funding for this work.

Competing interests: - The authors declare no competing interests

6. References

1. Lewison, R. (2007). Population responses to natural and human-mediated disturbances : assessing the vulnerability of the common hippopotamus (Hippopotamus amphibius). 407–415.
2. Kingdon, J. (2015). Kingdon field guide to African mammalian species-bloomsbury natural history (2nd ed.).
3. Cowdrey, D. (2019). *the state of the common hippo. july*
4. Furstenburg, D. (2018). Focus on the Hippopotamus (Hippopotamus amphibious) (Linnaeus ,

1758). *Geo Wild Consult, September*.

5. Eltringham, S. K. (1993). *Review of priorities for conservation action and future research on hippopotamuses. In: Pigs, Peccaries and HippopotamusStatus Survey Action Plan (1993) (Ed. by R. L.Oliver), Cambridge: IUCN. Pp. 127–148*.
6. Eltringham, S. K. (1999). *The Hippos: Natural History and Conservation. Poyser Natural History. Princeton University Press*. https://doi.org/10.1111/j.1474-7766.2005.00302.x
7. Kanga, E. M., Ogutu, J. O., Piepho, H. P., & Olff, H. (2013). Hippopotamus and livestock grazing: Influences on riparian vegetation and facilitation of other herbivores in the Mara Region of Kenya. *Landscape and Ecological Engineering*, *9*(1), 47–58. https://doi.org/10.1007/s11355-011-0175-y
8. Mulungu, L. S., Massawe, A. W., Kennis, J., Crauwels, D., Eiseb, S., Mahlaba, T. A., Monadjem, A., Makundi, R. H., Katakweba, A. A. S., Leirs, H., & Belmain, S. R. (2011). Differences in diet between two rodent species, Mastomys natalensis and Gerbilliscus vicinus, in fallow land habitats in central tanzania. *African Zoology*, *46*(2), 387–392. https://doi.org/10.3377/004.046.0218.
9. Hansson, L. (1999). *Nordic Society Oikos Intraspecific Variation in Dynamics : Small Rodents between Food and Predation in Changing Landscapes. Oikos 86:159-169*. *86*(1), 159–169.
10. Datiko, D., & Bekele, A. (2014). Habitat association and distribution of rodents and insectivores in Chebera Churchura National Park, Ethiopia. *Tropical Ecology*, *55*(2), 221–229.
11. Lewison, R. L., & Carter, J. (2004). Exploring behavior of an unusual megaherbivore : a spatially explicit foraging model of the hippopotamus. 171, 127–138. https://doi.org/10.1016/S0304-3800(03)00305-3
12. Chansa, W., Senzota, R., Chabwela, H., & Nyirenda, V. (2011). *The influence of grass biomass production on hippopotamus population density distribution along the Luangwa River in Zambia*. *January*.
13. Théophile, D. O., Sibiri, S., Issa, O., & Wendengoudi, G. (2012). Feeding habits of Hippopotamus amphibius and carrying capacity in the Biosphere Reserve of “Mare aux Hippopotames” in the South-Sudanian Zone of Burkina Faso. *Pakistan Journal of Zoology*, *44*(2), 433–442
14. Dudley, J. P., Hang’Ombe, B. M., Leendertz, F. H., Dorward, L. J., de Castro, J., Subalusky, A. L., & Clauss, M. (2016). Carnivory in the common hippopotamus Hippopotamus amphibius: Implications for the ecology and epidemiology of anthrax in African landscapes. Mammal Review, 46(3), 191–203. https://doi.org/10.1111/mam.12056
15. NMA, 2022. National Meteorological Agency, Addis Ababa, Ethiopia.
16. Martin, P. and Bateson, P. (2007). Measuring behaviour: An introductory guide. 3rd Edition. Cambridge University Press. Pp. 422. *Cambridge University Press.*, *32*(8), 898. https://doi.org/10.1016/0005-7967(94)90179-1
17. Timbuka, C. D. (2012). *The Ecology and Behaviour of the Common hippopotamus , Hippopotamus amphibious L . in Katavi National Park , Tanzania : Responses to Varying Water Resources Christopher David Timbuka Thesis submitted for the degree of Doctor of Philosophy at the Universit*. *July*.
18. Mekonnen, A. (2019). Ecology of Common Hippopotamus (Hippopotamus Amphibious, Linnaeus, 1758) and Conflict Incidence With Human around Chebera Churchura National Park, Ethiopia. *AddIis Ababa UNniversity, Addis Ababa, Ethiopia*

19. Landman, M. and Kerley, G. I. H. (2001). *Dietary shifts: do grazers become browsers in the thicket biome? Koedoe 44: 31–36.*

20. Macandza, V. A., Owen-Smith, N., & Cross, P. C. (2004). Forage selection by African buffalo in the late dry season in two landscapes. *African Journal of Wildlife Research*, *34*(2), 113–121.

21. Sierra, P. V., Cid, M. S., Brizuela, M. A., & Ferri, C. M. (2005). Microhistological estimation of grass leaf blade percentages in pastures and diets. *Rangeland Ecology and Management*, *58*(2), 207–214. https://doi.org/10.2111/1551-5028(2005)58<207:MEOGLB>2.0.CO;2

22. MacLeod, S. B., Kerley, G. I. H., & Gaylard, A. (1996). Habitat and diet of bushbuck Tragelaphus scriptus in the Woody Cape Nature Reserve: Observations from faecal analysis. *African Journal of Wildlife Research*, *26*(1), 19–25.

23.Katona, K., & Altbäcker, V. (2002). Diet estimation by faeces analysis: Sampling optimisation for the European hare. *Folia Zoologica*, *51*(1), 11–15.

24. Buys, D. (1990). Food selection by eland in the western Transvaal. S. Afr. J. Wildl. Res.20:16– 20.

25. Ellis, B. A., Russell, E. M., Dawson, T. J. and Harrop, C. J. F. (1977). Seasonal changes in diet preference of free-ranging red kangaroos, euros and sheep in western New South Wales. Aust. J. Wildl. Res. 4:127–144. *Journal of Zoology*.

26. Williams, O. B. (1969). An Improved Technique for Identification of Plant Fragments in Herbivore Feces. *Journal of Range Management*, *22*(1), 51.
https://doi.org/10.2307/3896035

27. Bartolomé, J., Franch, J., Plaixats, J., & Seligman, N. G. (1998). Diet selection by sheep and goats on Mediterranean heath-woodland range. *Journal of Range Management*, *51*(4), 383–391. https://doi.org/10.2307/4003322

28.Morrison, J. I. (2008). *Using Microhistological Techniques To Predict Botanical Composition of Horse Diets on Cool-Season Grass Pasture*. 1–73.

29. Prins, H. H. T., De Boer, W. F., Van Oeveren, H., Correia, A., Mafuca, J., & Olff, H. (2006). Co-existence and niche segregation of three small bovid species in southern Mozambique. *African Journal of Ecology*, *44*(2), 186–198. https://doi.org/10.1111/j.1365-2028.2006.00619.x

30. Mekonen, S., & Hailemariam, B. (2016). *Ecological behaviour of common hippopotamus (Hippopotamus amphibius , LINNAEUS , 1758) in boye wetland , jimma , ethiopia*. 41–49. https://doi.org/10.5251/ajsir.2016.7.2.41.49

31. Zubkowicz, R. (2005). *Selected problems of organising exhibition areas for common hippopotamus (Hippopotamus amphibius) - zoological data*. *218*(26), 211–218.

32.Saikawa, Y., Hashimoto, K., Nakata, M., Yoshihara, M., Nagai, K., Ida, M., & Komiya, T. (2004). "Pigment chemistry: the red sweat of the hippopotamus". Nature 42: 363–376. *Nature*, *429*(6990), 363. https://doi.org/10.1038/429363.

33.Fisher, R. E., Scott, K. M., & Naples, V. L. (2007). Forelimb myology of the pygmy hippopotamus (Choeropsis liberiensis). *Anatomical Record*, *290*(6), 673–693. https://doi.org/10.1002/ar.20531

34. Jones, R. (2008). *Husbandry Guidelines for the Common Hippopotamus Hippopotamus amphibius Mammalia : Hippopotamidae*.

35.Chritz, K. L., Blumenthal, S. A., Cerling, T. E., & Klingel, H. (2016). Hippopotamus (H. amphibius) diet change indicates herbaceous plant encroachment following

megaherbivore population collapse. *Scientific Reports*, *6*(February), 1–7. https://doi.org/10.1038/srep32807

36.Teshome, K., & Erena, M. G. (2022). Common hippopotamus in the upper Dhidhessa River and its conflicts with Arjo-Dhidhessa Sugar Factory, Ethiopia. *Conservation Science and Practice*, *4*(10), 1–12. https://doi.org/10.1111/csp2.12790

37.Noirard, C., Le Berre, M., Ramousse, R., & Lena, J. P. (2008). Seasonal variation of thermoregulatory behaviour in the Hippopotamus (Hippopotamus amphibius). *Journal of Ethology*, *26*(1), 191–193. https://doi.org/10.1007/s10164-007-0052-1

38. Gizachew, G., Tefera, Tessema, T. H., & Bekere, Tibebu Alemu Bekere, T. M. G. (2024). Human- common hippo (Hippopotamus amphibius) -conflict in the Dhidhessa Wildlife Sanctuary and its surrounding , Southwestern. PLoS ONE, 1–21. https://doi.org/10.1371/journal.pone.0303647

39. Gizachew, Tefera, T.H. Tessema, Tibebu Bekere, T.M.G. Population status and habitat suitability of the vulnerable common hippopotamus (Hippopotamus amphibius) in the Dhidhessa Wildlife Sanctuary, Southwestern Ethiopia. https://doi.org/10.1016/j.heliyon.2024.e40186.

Table 1: Diurnal activity and event records of male and female hippopotamus in the study area

	Behavioural activities					Total	Behavioural events		Total
Sex	Feeding	Resting	Moving	Standing	Socializing		Barking	Yawning	
Male	179	325	172	187	103	946	629	580	1209
Female	245	259	288	81	102	995	720	610	1330
Total	424	584	460	268	205	1941	1349	1190	2539
%	21.8	30.1	23.7	13.8	10.6	43.3	53.1	46.9	56.7

Table 2: Monthly variations of activities and events of male and female hippopotamus

	Behavioural activities						Behavioural events		
Months	Feeding	Resting	Moving	Standing	Socializing	Total	Barking	Yawning	Total
January	55	102	55	53	34	299	224	197	421
February	60	142	63	69	39	373	274	217	491
March	69	163	76	68	38	414	300	257	557
May	81	76	97	34	34	322	195	201	396
June	86	55	105	23	32	301	182	169	351
July	73	46	64	21	28	232	174	149	323
Total	424	584	460	268	205	1941	1349	1190	2539
(%)	21.8	30.1	23.7	13.8	10.6	43.3	53.1	46.9	56.7

Table 3: Plant species eaten by common hippopotamus in DWS

Family	Species	Frequency of observation	Percentage
Poaceae	*Echinochloa pyramidalis*	62	5.4
Poaceae	*Coelorhachis afraurita*	6	0.5
Poaceae	*Eriochloa fatmensis*	99	8.7
Poaceae	*Cynodon dactylon*	75	6.6
Poaceae	*Cynodon plectostachyus*	38	3.3
Poaceae	*Sacciolepis africana*	65	5.7
Poaceae	*Cymbopogon commutatus*	35	3.1
Poaceae	*Hyparrhenia glabriuscula*	30	2.6
Poaceae	*Cynodon nlemfuensis*	43	3.8
Poaceae	*Cymbopogon citratus*	37	3.3
Poaceae	*Hyparrhenia rufa*	19	1.7
Poaceae	*Hyparrhenia hirta*	27	2.4
Poaceae	*Hyparrhenia cymbaria*	7	0.6
Poaceae	*Panicum maximum*	5	0.4
Poaceae	*Oryza sativa*	3	0.3
Poaceae	*Echinochloa cruspavonis*	68	6
Poaceae	*Leptochloa rupestris*	56	4.9
Cyperaceae	*Cyperus rigidifolius*	83	7.3
Fabaceae	*Vigna vexillata*	5	0.4
Fabaceae	*Chamaeurista mimosoides*	3	0.3
Fabaceae	*Psoralea bituminosa*	2	0.2
Fabaceae	*Laburum anagyroide*	6	0.5
Onagraceae	*Epilobium hirsutum*	8	0.7
Commelinaceae	*Commelina benghalensis*	35	3.1
Amaranthaceae	*Alternanthera nodiflora*	67	5.9
Asteraceae	*Guizotia scabra*	8	0.7
Asteraceae	*Galinsoga parviflora cov.*	5	0.4
Apiaceae	*Hydrocotyle mannii*	42	3.7
Combretaceae	*Combretum pancultum*	6	0.5
Lamiaceae	*Orthosiphon schimperi*	31	2.7
Polygonaceae	*Fallopia baldschanira*	15	1.3
Polygonaceae	*Persicaria decipiens*	17	1.5
Polygonaceae	*Persicaria attenuate*	24	2.1
Typhaceae	*Typha latifolia*	107	9.4
	Total	1138	100

Table 4: Plant species consumed by hippos during the wet and dry seasons in DWS

Species	Wet season	Dry season	Total
Echinochloa pyramidalis	20 (2.3%)	40 (15.1%)	62 (5.4 %)
Coelorhachis afraurita	4 (0.5%)	2 (0.8%)	6 (0.5%
Eriochloa fatmensis	80 (9.2%)	19 (7.2%)	99 (8.7%)
Cynodon dacylon	60 (6.9%)	15 (5.7%)	75 (6.6%)
Cynodon plectostachyus	28 (3.2%)	10 (3.8%)	38 (3.3%)
Sacciolepis africana	56 (6.4%)	9 (3.4%)	65 (5.7%)
Cymbopogon commutatus	22 (2.5%)	13 (4.9%)	35 (3.1%)
Hyparrhenia glabriuscula	30 (3.4%)	-	30 (2.6%)
Cynodon nlemfuensis	23 (2.6%)	20 (7.5%)	43 (3.8%)
Cymbopogon citratus	21 (2.4%)	17 (6.4%)	37 (3.3%)
Hyparrhenia rufa	14(1.6%)	5 (1.9%)	19 (1.7%)
Hyparrhenia hirta	20 (2.3%)	7 (2.6%)	27 (2.4%)
Hyparrhenia cymbaria	5 (0.6%)	2 (0.8%)	7 (0.6%)
Panicum maximum	5 (0.6%)	-	5 (0.4%)
Oryza sativa	3 (0.3%)	-	3 (0.3%)
Echinochloa cruspavonis	50 (5.7%)	18 (6.8%)	68 (6%)
Leptochloa rupestris	45 (5.2%)	11 (4.2%)	56 (4.9%)
Cyperus rigidifolius	65 (7.4%)	18 (6.8%)	83 (7.3%)
Vigna vexillata	3 (0.3%)	2 (0.8%)	5 (0.4%)
Chamaeurista mimosoides	2 (0.2%)	1 (0.4%)	3 (0.3%)
Psoralea bituminosa	2 (0.2%)	-	2 (0.2%)
Laburum anagyroide	6 (0.7%)	-	6 (0.5%)
Epilobium hirsutum	-	8 (3%)	8(0.7%)
Commelina benghalensis	30 (3.4%)	5 (1.9%)	35 (3.1%)
Alternanthera nodiflora	67 (7.7%)	-	67 (5.9%)
Guizotia scabra	8 (0.9%)	-	8 (0.7%)
Galinsoga parviflora cov.	5 (0.6%)	-	5 (0.4%)
Hydrocotyle mannii	30 (3.4%)	12 (4.5%)	42 (3.7%)
Combretum pancultum	-	6 (2.3%)	6 (0.5%)
Orthosiphon schimperi	31 (3.6%)	-	31 (2.7%)
Fallopia baldschanira	11 (1.3%)	4 (1.5%)	15 (1.3%)
Persicaria decipiens	17 (1.9%)	-	17 (1.5%)

Persicaria attenuate	24(2.7%)	-	24 (2.1%)
Typha latifolia	86 (9.9%)	21 (7.9%)	107 (9.4%)
Total	873 (76.7%)	265 (23.3%)	1138 (100%)

Table 5: Mean percentage of food items identified from faecal analysis of hippopotamus in the study area

	Wet season				Dry season			
Samples	Graminoids	Herbs	Dicots	Unknown	Graminoids	Herbs	Dicots	Unknown
1	50	30	10	10	50	20	20	10
2	60	20	20	-	80	20	-	-
3	40	50	10	-	50	20	20	10
4	30	40	10	20	90	0	10	-
5	50	30	-	20	60	0	30	10
6	70	20	-	10	70	20	-	10
7	70	30	-	-	80	0	20	-
8	40	40	20	-	50	30	-	20
9	60	-	30	10	60	10	10	20
10	50	30	20	-	80	10	10	-
11	60	30	-	10	90	0	-	10
12	40	50	10	-	70	20	10	-
13	70	30	-	-	30	30	30	10
14	80	20	-	-	70	30	-	-
15	50	30	20	-	40	30	20	10
16	80	20	-	-	60	0	30	10
17	60	-	30	10	50	30	20	-
18	50	40	-	10	60	40	-	-
19	30	60	10	-	70	0	20	10

20	50	20	20	10	80	20	-	-
Mean	54.5	29.5	10.5	5.5	64.5	16.5	12.5	6.5

Manuscript

Click here to access/download;Manuscript;manuscript with anonymos.docx

Feeding ecology and activity patterns of Common Hippopotamus in the Dhidhessa Wildlife Sanctuary, Southwestern Ethiopia

Abstract

The study of activity patterns and dietary composition for hippos is essential for understanding the links between this species and the environment in which it lives. The objective of this research was to examine the activity patterns and dietary habits of common hippopotamuses in the Dhidhessa Wildlife Sanctuary (DWS) southwest, Ethiopia. The survey was carried out between 2022 and 2023. Event patterns and behavioral activity were studied with 30-minute intervals following a male and female as focal sampled individuals. In order to study the hippopotamus' diet, plant species identified from fresh eating signals were gathered, and supplemented by fecal analysis. With 30.1% of their time spent resting, they made up the biggest amount of all behavioral activities, followed by moving (23.7%). 53.1% of their time was spent barking, while 46.9% was spent yawning. Compared to females, males rested more. With a resting peak in the middle of the day, feeding and movement peak activities were noted in the early morning and late afternoon. Both male and female hippopotamuses exhibited a greater frequency of barking and yawning in the afternoon. Thirty-four plant species from twelve families were devoured by hippos. Family Poaceae contributed the largest (60%), proportion of their diet while Combretaceae is the least (0.5%). Based on the overall percentage contribution, Typha latifolia was the most consumed plant species, which accounted for 9.4% followed by Eriochloa fatmensis (8.7%) while, Oryza sativa and Chamaeurista mimosoides (0.3%, for each). Therefore, DWS's primary priority should be creating management plans to reestablish the sanctuary as a fully functional sustainable ecosystem.

Keywords: Behavioural activity, Common Hippopotamus, Dhidhessa Wildlife Sanctuary, Feeding ecology.

Introduction

Background of the study

The common hippopotamus, *Hippopotamus amphibius L*, (hereafter referred to as hippo), was once widespread across wet habitats throughout Africa [1], particularly in lakes and rivers of sub-Saharan Africa [2] . In terms of size and weight, it ranks third among terrestrial mammals, behind white rhinos and elephants [3] . The recognized species of hippo is distinguished by its large, round head, short bulky limbs, broad nose, short tail, short stocky legs, and very long tusk-like canines. They have eyes, ears, and nostrils on the high roof of the cranium [4] . Due to their thin epidermis, hippos are prone to desiccation. The natural sunscreen chemical "blood sweat" that their skin secretes helps shield them from the sun. Despite this, because they lack sweat glands, their skin will fracture if exposed to air for too long[5] .

Hippos are considered obligatory herbivores, meaning they favor plants and short grass [6,7]. Animal evolution, life-history techniques, and ecological role are all shaped by their diets [8] . Food is one of the niche's most important dimensions. As a result, most ecological studies require knowledge of animal diets. The study of animal diets is essential for understanding the links between species and the environment in which they live [9,10]. These relationships may determine community structure, species diversity, relative abundance, and resource partitioning among species and individuals.

As the name implies, the *Hippopotamus amphibius* is an amphibious animal with two primary habitat needs: grass for nighttime grazing and water for daytime repose [11]. Hippos can be found in a variety of permanent freshwater environment, including ponds, rivers, lakes, and waterholes with gently sloping banks surrounded by grazing areas. It avoids fast-moving streams

with heavily swampy vegetation and rocky outcroppings [1]. The hippos are a selective grazer that consumes 35–50 kg of grass per day, or roughly 2.5% of its body weight [12, 13]. Unlike other mega-herbivores, it needs both an open grazing range and a place to live in water on a daily basis [5].

The research on the ecology and behavior of hippos, as well as the invasive and often antagonistic relationship between humans and hippos, is still lacking [14]. These points have led to the formulation of the objective of this study. The intended purposes for which the DWS, Southwestern Ethiopia, was formed have not been met recently. Large mammal populations in the vicinity subsequently moved into the adjacent forest patches. For one thing, the DWS is home to a variety of large mammals, including hippopotamuses. Hence, the current study area focused on the feeding ecology and behavioral activity of the common hippo in order to develop long-term conservation measures in the study area, all of which will help to establish a national park in the area.

Materials and methods

Ethical statement

The research proposal (supplementary file) was reviewed and granted approval by the Review Board at Jimma University (JU) College of Agriculture, Veterinary, and Medicine. The vice president's office also reauthorized and accepted the request for research and community service from the review board. JU sanctioned the process for collecting consent needed for the data collection tools (RGS/752/2021). Consent to participation is not applicable since no respondents were participated in this research work. All methods used in this study were performed in accordance with the relevant guidelines and regulations.

Description of the Study Area

The study was conducted in the DWS and its surrounding areas in southwestern Ethiopia, spanning three administrative zones: Jimma, Buno Bedele, and East Wollega, all within the Oromia National Regional State. The total area is approximately 1300 km². It is situated 395 km West of Addis Ababa, along the Addis Ababa Nekemte-Bedele route. Geographically, the location is between 8^{0}30’0” and 8^{0}40’0” N latitude and 36^{0}22’0” and 36^{0}43’ 0” E longitude (Fig.1 our team used shape files provided by the Ethiopian Mapping Agency to produce the map. The agency’s official website offers free access to these shape files (http://www.ema.gov.et). The altitude in the region varies from 1050 to 2560 meters. The area receives annual precipitation in a uni-modal pattern, ranging from 648 to 2001.8 mm. The Sanctuary has two distinct seasons: a dry season from November to February and a wet season from June to September. The lowland areas within the sanctuary tend to have warmer temperatures, with average minimum and maximum annual temperatures of 12°C and 35°C, respectively [15]. The predominant vegetation types in the study area include *Combretum*, *Commiphora*, and *Acacia* species, interspersed with a primarily growing savanna grass composed of *Hyparrhenia* species.

Methods

In order to record the activity patterns of hippos in the study area, a focal animal sampling method was used, focusing on two focal sampled animals were selected using the methodology suggested by [17] in this same area Data on the daytime behaviors of a pair of focal animals—an adult male and an adult female—were recorded every 30 minutes, with a 5-minute sampling interval in between. Focal animals were selected randomly and clearly identified by distinctive morphological characteristics (skin mark or skin colour) by using binoculars or naked eyes. Additionally, a group or individual hippos was rapidly scanned by using camera traps at the

sampling location, which were scanned at five-minute intervals every 24 hours during the dry (January to March) and rainy seasons (May to July), and the behavior of each animal was recorded at that instant. Observations were conducted for five consecutive days in each of the six months. A total of 120 days of behavioural observations were done in both seasons. During each season, behavioral activity was recorded as resting, standing, walking, feeding, and socializing, while yawning and barking were recorded as behavioral events every five minutes (Table S1 and S2). These behavioral categories, however, were referred to as behavioral actions and occurrences [17, 18]. [16] Assert that behavioral events are a shorter period of time, whereas behavioral activities are long-duration.

Based on information from a preliminary survey in the study area, the plant species that hippos eat were investigated by looking at new feeding indicators following focal groups at close distances [13]. The food plants the animal had bitten or plucked were collected, compressed, and identified with the help of a taxonomist from the Botanical Herbarium at Wollega University. To calculate a plant species' percentage of feeding preference, the frequency of biting or feeding sign observations for that species were added up, divided by the total frequency of all plant species gathered at feeding sites, and then multiplied by 100 over the course of the study, $\mathrm{CSi} = \frac{FSi}{\sum_{i=1}^{n} FSt} x100$, where FSi is the frequency of species i, which corresponds to the number of hits of the species along transects within a plot CSi, the contribution of species i to the sum of the frequencies of all species; it reflects the abundance of a species and expressed as a percentage and $\sum_{i=1}^{n} \mathrm{FSt} =$ the total frequency of all collected species.

The fecal analysis method was used in conjunction with the feeding observation technique to identify species that were foraged [19-21]. Dung samples were gathered from various habitats and air-dried prior to analysis [22] (Figure S1). Fecal samples collected during the same season

were combined to create composite samples [23] These composite samples were manually broken apart and thoroughly washed with water using a 0.1 mm sieve to eliminate finely digested food particles [24] . Subsequently, samples were placed into a beaker, treated with 75% alcohol, and allowed to sit for 48 hours. After the alcohol was decanted, domestic bleach was introduced and left to react until the bleaching was complete [26, 26]. The samples were then rinsed with water, stained with safranin, and allowed to sit for 2 days [25] prior to being examined microscopically. Ten slides from each composite fecal sample were analyzed using a compound light microscope. A small portion of the prepared fecal sample was applied to a gridded slide and counted under ×100 and × 40 magnifications [27]. The counting and identification of epidermal fragments were supported by reference slides [28] and by examining morphological characteristics such as cell shape, venation, and trichomes [28]. Species-level identified fragments were categorized into graminoids, herbs, and dicots [22, 29], while those that could not be identified were classified as unknown.

Results

Diurnal activity and event patterns of hippopotamus

A total of 4,480 behavioral records were collected from adult male and female common hippos through focal sampling. Among these records, 43.3% (1,941) were categorized as activities, while the remaining 56.7% (2,539) were events documented during a total of 540 hours of behavioral observation. Furthermore, the observational data indicated that hippos spent a significantly greater proportion of their time in resting activities (30.1%) compared to other types of activities (see Table 1). However, barking and yawning events constituted 53.1% and 46.8% of the observed behaviors, respectively (Figure S2).

Time-specific activity budgets

Certain aspects of the activity budget for both male and female hippopotamuses exhibited notable variations throughout the day. The behaviors of feeding, moving, resting, and standing changed significantly at different times over the course of the study (Fig. 2). Feeding, moving, and standing behaviors each displayed two peaks: one in the early morning hours from 07:00 to 09:00 and another in the late afternoon from 16:00 to 18:00. The behaviors of resting or lying down were particularly prominent during the hottest parts of the day, specifically from 11:00 to 15:00. The final activity noted for both male and female hippopotamuses was socialization, which included mating behaviors. Instances of barking and yawning were recorded throughout the day in both sexes, with a marked increase in the afternoon. The yawning behavior involves the animal raising its head and extending its mouth forward (Fig. 3). After yawning, they tend to open and close their mouths repeatedly while emitting low grunts and engaging in more head bobbing. The yawning behaviors of male and female hippopotamuses were found to be quite similar.

Monthly variations in activity and event budget

The highest frequency of feeding occurred in June, followed by May, while January and February had the lowest feeding occurrences (Table 2). June also recorded the greatest amount of movement. The levels of movement in February and July were relatively similar. March had the highest resting figures, with February following, while January and July recorded the least moving and standing, respectively. The peak social interaction among hippos was noted in February and March, whereas July saw the lowest socializing levels. March had the most frequent barking events, with February in second place, while July had the fewest incidences of barking. The most yawning among hippos was observed in March, followed by February, whereas July had the least yawning, closely followed by May (Table 2).

Comparison in activity and event budget

The distribution of time that adult females and males spent on various activities and events throughout the study period as displayed in Table 1. Among the 1941 and 2539 behavioral activities and events recorded, male hippopotamuses exhibited a lower percentage (48.7% and 47.6%) of these activities compared to females (51.3% and 52.4%). Females engage in more behavioral activities than their male counterparts.

During the daytime, male hippos devoted 32.2% of their activity to resting. The next most common activity was standing, which accounted for 19.8%, followed by feeding at 18.9% (refer to Figure S3). In terms of the daytime activities of female hippos, the highest recorded activities were moving (28.9%) and resting (28%), with feeding following closely at 24%. However, there were no significant differences observed in the activity patterns between male and female hippos ($t=2.18$, $df= 6$, $P>0.05$) (Fig.4).

Seasonal variations in activity budget

During the wet season, there were a total of 1110 recorded behavioral activities, while the dry season accounted for 831. Behavioral events were documented in both the rainy (1070) and dry (1469) seasons. In the dry season, hippopotamuses allocated their time to resting (37.1%), standing (17.8%), and barking (54.3%), with these activities being more pronounced compared to the wet season. Conversely, in the wet season, the hippos showed a higher frequency of feeding (28.9%), walking (31.8%), and yawning (48.5%) when compared to the dry season (Fig.5). Significant differences were observed in the activity budgets between the dry and wet seasons for hippos ($t=2.44$, $df =6$, $p<0.05$).

Feeding ecology

A total of 34 plant species from 12 families (excluding crops) were identified (Table 3). Among these, 23 species were documented through observations of feeding signs and daytime feeding records during focal sampling, while the other 11 species were noted based on observed feeding bites. The Poaceae family made the largest contribution to their diet, constituting 60%, whereas Combretaceae had the smallest share at 0.5% (Figure S4). In terms of overall percentage contribution, *Typha latifolia* emerged as the most consumed plant species, making up 9.4%, followed closely by *Eriochloa fatmensis* at 8.7%. Meanwhile, *Oryza sativa* and *Chamaeurista mimosoides* each represented 0.3%, with *Psoralea bituminosa* following at 0.2% (Table 3).

Alongside the species present in the DWS, crops such as Maize (*Zea mays*), Sugar cane (*Saccharum officinarum*), Banana (*Musa paradisiaca*), Great millet (*Sorghum bicolor*), Garlic (*Allium sativum*), and Onion (*Allium cepa*) were significantly consumed by the hippopotamus in agricultural areas.

During the wet season, hippos were observed eating a wider range of forage species (76.7%) in contrast to the dry season (23.3%). The most frequently eaten forage species by hippos in the rainy season included *Typha latifolia* (9.9%), *Eriochloa fatmensis* (9.2%), *Alternanthera nodiflora* (7.7%), *Cyperus rigidifolius* (7.4%), and *Cynodon dacylon* (6.9%). In contrast, the dry season's predominantly consumed plant species were *Echinochloa pyramidalis* (15.1%), *Typha latifolia* (7.9%), *Cynodon nlemfuensis* (7.5%), *Eriochloa fatmensis* (7.2%), along with *Echinochloa cruspavonis* and *Cyperus rigidifolius* (both at 6.8%). Nonetheless, during the wet season, the least frequently consumed forage species included *Vigna vexillata* and *Oryza sativa* (0.3% for each), and *Chamaeurista mimosoides* and *Psoralea bituminosa* (0.2% for each), while the dry season saw *Chamaeurista mimosoides* (0.4%) and *Hyparrhenia cymbaria*, *Coelorhachis*

afraurita, and *Vigna vexillata* (0.8% for each) as the lowest consumed forage species in the study area. Importantly, there were notable differences in the consumption of each plant species across the various seasons throughout the study period ($t = 2.03$, $df = 33$, $P<0.05$) (Table 4).

An examination of 80 hippopotamus droppings showed that different quantities of dietary elements, such as dicots, grasses (graminoids), forbs (herbs), and unidentified substances, were ingested during both the wet and dry seasons (Table 5).

Discussion

Activity pattern

Examining the fundamental behaviors of hippopotamuses is essential for evaluating the well-being of hippo populations, their ecosystems, and the potential for lasting coexistence with local communities [6, 17]. The results of the present study carried out in DWS indicated that hippos participated in a range of activities during the day, primarily resting, along with walking, feeding, standing, and engaging in social interactions. This observation aligns with the results reported by [18] in Chebera Churchura National Park and [30] in Boye Wetland, Ethiopia. Conversely, the current study's results differ from [17], who noted that hippos rest for over half the day. This discrepancy may be attributed to the differing sampling methods, as this study utilized focal sampling while Timbuka employed scan sampling. Nevertheless, there were variations in behavioral events like barking and yawning among hippopotamuses.

The current study's findings revealed that the hippopotamus exhibits a general activity pattern marked by peaks in the morning and late afternoon, with a resting phase during midday. In the morning, they devoted several hours to feeding, frequently returning to the water, which made them somewhat agitated. During the daytime heat, most hippopotamuses were observed resting.

This behavior serves as a crucial adaptation for their survival and helps prevent their skin from drying out under the sun [31]. Around midday, the hippopotamus typically refrained from feeding, except on overcast or rainy days. A similar observation was made by [17, 18, 30], who noted that hippopotamuses often move and enter the water frequently during hot weather. The results of this study revealed that hippopotamuses did not entirely leave the water. This observation corresponds with studies carried out by [6,18,30,32, which reported that most of their bodies remained submerged while they extended their necks to graze on grasses floating on the surface or growing at the water's edge.

The current study's findings revealed that the hippopotamus exhibits a general activity pattern marked by peaks in the morning and late afternoon, with a resting phase during midday. In the morning, they devoted several hours to feeding, frequently returning to the water, which made them somewhat agitated. During the daytime heat, most hippopotamuses were observed resting. This behavior serves as a crucial adaptation for their survival and helps prevent their skin from drying out under the sun [31]. Around midday, the hippopotamus typically refrained from feeding, except on overcast or rainy days. A similar observation was made by [17, 18, 30], who noted that hippopotamuses often move and enter the water frequently during hot weather. The findings of the current study indicated that hippopotamuses did not completely exit the water. This finding aligns with research conducted by [6,18, 30,32], who noted that, most part of their body submersed in the water and extends the neck to the grasses that fluted on the water or grow at the edge parts have to forage.

The current study in DWS indicated that the activity budget percentage of hippopotamuses displayed variations throughout the months. This could be linked to factors such as the availability of a consistent water source, favored plant species near their resting areas, and

environmental temperatures, which significantly influenced the hippopotamus's activity patterns. Feeding activity was most prevalent in June, followed by May, and while it was least observed in February and March. During February and March, the temperatures rise and rainfall and cloud cover are minimal. As a result, hippopotamuses tended to rest during the day, often fully submerged in water or moving to shaded spots, and generally had less need to feed. Conversely, in June, the weather became cloudier, prompting increased activity as they searched for food and moved around. Feeding activity during the day also diminished in July, possibly because they had secured enough tender grass at night and faced reduced competition from livestock, leading to a decline in both feeding and resting activities during that month. The results of this research correspond with the outcomes of [18,30,33], who noted that increased water levels and flooding affect the animals' movements in search of food, causing the grass to submerge in water. Nonetheless, a notable increase in barking and yawning was recorded in March. Comparable results were observed by [6,18, 30], who observed that during March and April, hippopotamuses exhibited the highest frequency of yawning and barking, likely related to mating behavior.

Research indicates that the behavioral differences between male and female hippopotamuses may be influenced by various factors, including disturbance, hunger, play, aggression, and mating activities. The results of the current study affirm that females engage in more behavioral activities than males. This result aligns with the observations made by [6], who noted that females exhibited greater aggression and vocalization when defending their young from threats posed by males or other disturbances. The female hippo fiercely guards her calf and displays behaviors such as barking and yawning. Conversely, both male and female hippos exhibit barking and yawning behaviors at any time throughout the day, with a notable increase during the afternoon. Similar observations were documented by [12, 18, and 30]. The seasonal patterns

of certain species may affect their behavioral variations across different seasons. The findings of the current study support the notion that during the dry season, hippos primarily engage in resting and standing, while the rainy season is associated with heightened rates of feeding and movement. Furthermore, due to competition and mating periods, barking and yawning behaviors were particularly prominent during the dry months. Similar observations were reported by [17, 34].

Feeding ecology

Research into the feeding habits of hippos is quite sparse, with many previous studies primarily focusing on qualitative data rather than quantitative analysis [35]. A diet survey of hippos conducted in the DWS and surrounding areas during this study found that they consumed 34 different plant species in both wet and dry seasons. This finding aligns with the research by [13] conducted in the Biosphere Reserve of Burkina Faso, which also identified 34 plant species, and [18] in Chebra Churchura National Park, Ethiopia, which noted 40 species. The higher number of species recorded in the study from Chebra Churchura National Park may be attributed to its well-preserved biodiversity with minimal human disruption, allowing for a wider range of plant species to be accessible to hippos. Conversely, the current study identified more plant species consumed by hippos compared to the research done by [36] in the Arjo Dhidhessa sugar factory, Ethiopia; [30] in the Boye Wetland, Ethiopia; and the Luangwa River in Zambia [12]. The disparity in the quantity of plant species eaten by hippos in this study area compared to earlier research may be influenced by the duration of the study periods and the decline in species diversity caused by human activities such as settlement expansion and agriculture. This finding is consistent with earlier studies conducted by [13] and [37]. In this study, various plant species were consumed by hippos, with *T. latifolia*, *E. fatmensis*, *C. rigidifolius*, *C. dactylon*, and

Sacciolepis africana being the most favored and constituting the largest part of their diet. Additionally, *C. dactylon* has frequently been identified as a primary food source for hippos in various nations. A comparable finding has been recorded by[12,18,36] .

The findings of this research revealed that almost all plant species were eaten by hippos during both seasons, except for *Alternanthera nodiflora*, *Orthosiphon schimperi*, *P. attenuato*, *Persicaria decipiens*, *Hyparrhenia glabriuscula*, *Panicum maximum*, *Oryza sativa*, *Psoralea bituminosa*, *Laburum anagyroide*, *Alternanthera nodiflora*, *Guizotia scabra*, and *Galinsoga parviflora*, which were not present in the dry season. Nevertheless, this suggests that a greater variety of species was consumed in the wet season compared to the dry season. As the dry season advanced, the diversity of species eaten by hippos declined. This alteration in the hippo's dietary composition may be attributed to the seasonal availability of various grass and herbaceous species in the study area. This observation aligns with the findings of [18, 36]. The types of forage eaten by hippos were divided into three groups: grasses, herbs, and shrubs. Of these, grasses made up the most significant part of the hippo's diet, comprising 66% in the study region. Numerous studies conducted in other locations have indicated that the feeding habits of hippos are predominantly comprised of grasses [5,6]. In the current study, the primary grasses from the Poaceae and Cyperceae families were significantly grazed during both the dry and wet seasons. The outcomes of the current research are consistent with the results of earlier studies conducted by [18, 36].

Conclusion

According to the results of this study, hippos have effectively adapted to thrive in DWS and the surrounding areas of southwestern Ethiopia. Among the various behavioral activities observed,

resting constituted the largest share for both males and females. Females displayed greater activity compared to males in movements, barking, and yawning. In contrast, males dedicated more time to resting than their female counterparts. Apart from social interactions, females were overall more active than males regarding their behaviors and activity patterns. Hippo activity levels peaked during the morning and late afternoon, while they tended to rest during the midday hours. There were variations in activity components and event patterns across different months and seasons. The dietary preferences of hippos reflect their ability to adapt to their surroundings, as they feed on a variety of plant species such as grasses, shrubs, and herbs. Habitats rich in vegetation and water are particularly favorable for hippos and other animals in the region. Although the forest environment remains largely intact and closed, the increasing human population, along with pressures from large herbivores, illegal settlements, agricultural expansion, and conflicts between humans and hippos (and other wildlife) could lead to excessive exploitation of limited resources in the area [38]. DWS has significant potential to support a considerable number of hippos and other wildlife, which could be developed into an ecotourism hub [39]. Therefore, it is strongly suggested that the Ethiopian Wildlife Authority take steps to reestablished the sanctuary designation of DWS and consider elevating it to national park status, while implementing necessary conservation measures, such as community-based conservation initiatives, to address existing issues. A thorough assessment of biodiversity in the area should be conducted to establish an effective long-term conservation and management strategy for hippos and other wildlife present.

Supporting information

Table S1: Data sheet used for behavioural activity and event patterns (daily cycle)

Table S2: Data sheet used for behavioural activity and event patterns by adult hippo (monthly).

Figure S1: Images illustrating faecal analysis in microscopes at the laboratory of biology department of Wollega University.

Figure S2: Distribution and different behavioural activities of hippo in the DWS.

Figure S3: Some of the grass species foraged by hippopotamus in DWS.

Acknowledgements: We thank to IDEA-WILD for their material support. The authors would like to acknowledge Arjo Dhidhessa Sugar Factory, local communities. And district experts who assisted the field survey. Also, they would like to thank Ashetu Kejela for preparing the map of the study area and Mokonnen Teshome for Botanical Herbarium, Wollega University and for diet quality analysis.

Data Availability: All relevant data is within the paper and it's supporting information files.

Funding: The author(s) received no specific funding for this work.

Competing interests: - The authors declare no competing interests

6. References

1. Lewison, R. (2007). Population responses to natural and human-mediated disturbances : assessing the vulnerability of the common hippopotamus (Hippopotamus amphibius). 407–415.
2. Kingdon, J. (2015). Kingdon field guide to African mammalian species-bloomsbury natural history (2nd ed.).
3. Cowdrey, D. (2019). *the state of the common hippo. july*
4. Furstenburg, D. (2018). Focus on the Hippopotamus (Hippopotamus amphibious) (Linnaeus , 1758). *Geo Wild Consult, September*.
5. Eltringham, S. K. (1993). *Review of priorities for conservation action and future research on hippopotamuses. In: Pigs, Peccaries and HippopotamusStatus Survey Action Plan (1993) (Ed. by R. L.Oliver), Cambridge: IUCN. Pp. 127–148*.
6. Eltringham, S. K. (1999). *The Hippos: Natural History and Conservation. Poyser Natural History. Princeton University Press.* https://doi.org/10.1111/j.1474-7766.2005.00302.x
7. Kanga, E. M., Ogutu, J. O., Piepho, H. P., & Olff, H. (2013). Hippopotamus and livestock grazing: Influences on riparian vegetation and facilitation of other herbivores in the Mara Region of Kenya. *Landscape and Ecological Engineering*, *9*(1), 47–58. https://doi.org/10.1007/s11355-011-0175-y
8. Mulungu, L. S., Massawe, A. W., Kennis, J., Crauwels, D., Eiseb, S., Mahlaba, T. A., Monadjem, A., Makundi, R. H., Katakweba, A. A. S., Leirs, H., & Belmain, S. R.

(2011). Differences in diet between two rodent species, Mastomys natalensis and Gerbilliscus vicinus, in fallow land habitats in central tanzania. *African Zoology*, *46*(2), 387–392. https://doi.org/10.3377/004.046.0218.

9. Hansson, L. (1999). *Nordic Society Oikos Intraspecific Variation in Dynamics : Small Rodents between Food and Predation in Changing Landscapes. Oikos 86:159-169*. *86*(1), 159–169.

10. Datiko, D., & Bekele, A. (2014). Habitat association and distribution of rodents and insectivores in Chebera Churchura National Park, Ethiopia. *Tropical Ecology*, *55*(2), 221–229.

11. Lewison, R. L., & Carter, J. (2004). Exploring behavior of an unusual megaherbivore : a spatially explicit foraging model of the hippopotamus. 171, 127–138. https://doi.org/10.1016/S0304-3800(03)00305-3

12. Chansa, W., Senzota, R., Chabwela, H., & Nyirenda, V. (2011). *The influence of grass biomass production on hippopotamus population density distribution along the Luangwa River in Zambia*. *January*.

13. Théophile, D. O., Sibiri, S., Issa, O., & Wendengoudi, G. (2012). Feeding habits of Hippopotamus amphibius and carrying capacity in the Biosphere Reserve of “Mare aux Hippopotames” in the South-Sudanian Zone of Burkina Faso. *Pakistan Journal of Zoology*, *44*(2), 433–442

14. Dudley, J. P., Hang’Ombe, B. M., Leendertz, F. H., Dorward, L. J., de Castro, J., Subalusky, A. L., & Clauss, M. (2016). Carnivory in the common hippopotamus Hippopotamus amphibius: Implications for the ecology and epidemiology of anthrax in African landscapes. Mammal Review, 46(3), 191–203. https://doi.org/10.1111/mam.12056

15. NMA, 2022. National Meteorological Agency, Addis Ababa, Ethiopia.

16. Martin, P. and Bateson, P. (2007). Measuring behaviour: An introductory guide. 3rd Edition. Cambridge University Press. Pp. 422. *Cambridge University Press.*, *32*(8), 898. https://doi.org/10.1016/0005-7967(94)90179-1

17. Timbuka, C. D. (2012). *The Ecology and Behaviour of the Common hippopotamus , Hippopotamus amphibious L . in Katavi National Park , Tanzania : Responses to Varying Water Resources Christopher David Timbuka Thesis submitted for the degree of Doctor of Philosophy at the Universit*. *July*.

18. Mekonnen, A. (2019). Ecology of Common Hippopotamus (Hippopotamus Amphibious, Linnaeus, 1758) and Conflict Incidence With Human around Chebera Churchura National Park, Ethiopia. *AddIis Ababa UNniversity, Addis Ababa, Ethiopia*

19. Landman, M. and Kerley, G. I. H. (2001). *Dietary shifts: do grazers become browsers in the thicket biome? Koedoe 44: 31–36.*

20. Macandza, V. A., Owen-Smith, N., & Cross, P. C. (2004). Forage selection by African buffalo in the late dry season in two landscapes. *African Journal of Wildlife Research*, *34*(2), 113–121.

21. Sierra, P. V., Cid, M. S., Brizuela, M. A., & Ferri, C. M. (2005). Microhistological estimation of grass leaf blade percentages in pastures and diets. *Rangeland Ecology and Management*, *58*(2), 207–214. https://doi.org/10.2111/1551-5028(2005)58<207:MEOGLB>2.0.CO;2

22. MacLeod, S. B., Kerley, G. I. H., & Gaylard, A. (1996). Habitat and diet of bushbuck Tragelaphus scriptus in the Woody Cape Nature Reserve: Observations from faecal analysis. *African Journal of Wildlife Research*, *26*(1), 19–25.

23.Katona, K., & Altbäcker, V. (2002). Diet estimation by faeces analysis: Sampling optimisation for the European hare. *Folia Zoologica*, *51*(1), 11–15.
24. Buys, D. (1990). Food selection by eland in the western Transvaal. S. Afr. J. Wildl. Res.20:16– 20.
25. Ellis, B. A., Russell, E. M., Dawson, T. J. and Harrop, C. J. F. (1977). Seasonal changes in diet preference of free-ranging red kangaroos, euros and sheep in western New South Wales. Aust. J. Wildl. Res. 4:127–144. *Journal of Zoology*.
26. Williams, O. B. (1969). An Improved Technique for Identification of Plant Fragments in Herbivore Feces. *Journal of Range Management*, *22*(1), 51.
https://doi.org/10.2307/3896035
27. Bartolomé, J., Franch, J., Plaixats, J., & Seligman, N. G. (1998). Diet selection by sheep and goats on Mediterranean heath-woodland range. *Journal of Range Management*, *51*(4), 383–391. https://doi.org/10.2307/4003322
28.Morrison, J. I. (2008). *Using Microhistological Techniques To Predict Botanical Composition of Horse Diets on Cool-Season Grass Pasture*. 1–73.
29. Prins, H. H. T., De Boer, W. F., Van Oeveren, H., Correia, A., Mafuca, J., & Olff, H. (2006). Co-existence and niche segregation of three small bovid species in southern Mozambique. *African Journal of Ecology*, *44*(2), 186–198. https://doi.org/10.1111/j.1365-2028.2006.00619.x
30. Mekonen, S., & Hailemariam, B. (2016). *Ecological behaviour of common hippopotamus (Hippopotamus amphibius , LINNAEUS , 1758) in boye wetland , jimma , ethiopia*. 41–49. https://doi.org/10.5251/ajsir.2016.7.2.41.49
31. Zubkowicz, R. (2005). *Selected problems of organising exhibition areas for common hippopotamus (Hippopotamus amphibius) - zoological data*. *218*(26), 211–218.
32.Saikawa, Y., Hashimoto, K., Nakata, M., Yoshihara, M., Nagai, K., Ida, M., & Komiya, T. (2004). “Pigment chemistry: the red sweat of the hippopotamus”. Nature 42: 363–376. *Nature*, *429*(6990), 363. https://doi.org/10.1038/429363.
33.Fisher, R. E., Scott, K. M., & Naples, V. L. (2007). Forelimb myology of the pygmy hippopotamus (Choeropsis liberiensis). *Anatomical Record*, *290*(6), 673–693. https://doi.org/10.1002/ar.20531
34. Jones, R. (2008). *Husbandry Guidelines for the Common Hippopotamus Hippopotamus amphibius Mammalia : Hippopotamidae*.
35.Chritz, K. L., Blumenthal, S. A., Cerling, T. E., & Klingel, H. (2016). Hippopotamus (H. amphibius) diet change indicates herbaceous plant encroachment following megaherbivore population collapse. *Scientific Reports*, *6*(February), 1–7. https://doi.org/10.1038/srep32807
36.Teshome, K., & Erena, M. G. (2022). Common hippopotamus in the upper Dhidhessa River and its conflicts with Arjo-Dhidhessa Sugar Factory, Ethiopia. *Conservation Science and Practice*, *4*(10), 1–12. https://doi.org/10.1111/csp2.12790
37.Noirard, C., Le Berre, M., Ramousse, R., & Lena, J. P. (2008). Seasonal variation of thermoregulatory behaviour in the Hippopotamus (Hippopotamus amphibius). *Journal of Ethology*, *26*(1), 191–193. https://doi.org/10.1007/s10164-007-0052-1
38. Gizachew, G., Tefera, Tessema, T. H., & Bekere, Tibebu Alemu Bekere, T. M. G. (2024). Human- common hippo (Hippopotamus amphibius) -conflict in the Dhidhessa Wildlife Sanctuary and its surrounding , Southwestern. PLoS ONE, 1–21. https://doi.org/10.1371/journal.pone.0303647

39. Gizachew, Tefera, T.H. Tessema, Tibebu Bekere, T.M.G. Population status and habitat suitability of the vulnerable common hippopotamus (Hippopotamus amphibius) in the Dhidhessa Wildlife Sanctuary, Southwestern Ethiopia. https://doi.org/10.1016/j.heliyon.2024.e40186.

Table 1: Diurnal activity and event records of male and female hippopotamus in the study area

	Behavioural activities					Total	Behavioural events		Total
Sex	Feeding	Resting	Moving	Standing	Socializing		Barking	Yawning	
Male	179	325	172	187	103	946	629	580	1209
Female	245	259	288	81	102	995	720	610	1330
Total	424	584	460	268	205	1941	1349	1190	2539
%	21.8	30.1	23.7	13.8	10.6	43.3	53.1	46.9	56.7

Table 2: Monthly variations of activities and events of male and female hippopotamus

	Behavioural activities						Behavioural events		
Months	Feeding	Resting	Moving	Standing	Socializing	Total	Barking	Yawning	Total
January	55	102	55	53	34	299	224	197	421
February	60	142	63	69	39	373	274	217	491
March	69	163	76	68	38	414	300	257	557
May	81	76	97	34	34	322	195	201	396
June	86	55	105	23	32	301	182	169	351
July	73	46	64	21	28	232	174	149	323
Total	424	584	460	268	205	1941	1349	1190	2539
(%)	21.8	30.1	23.7	13.8	10.6	43.3	53.1	46.9	56.7

Table 3: Plant species eaten by common hippopotamus in DWS

**Family**	**Species**	**Frequency of observation**	**Percentage**
Poaceae	*Echinochloa pyramidalis*	62	5.4
Poaceae	*Coelorhachis afraurita*	6	0.5
Poaceae	*Eriochloa fatmensis*	99	8.7
Poaceae	*Cynodon dactylon*	75	6.6
Poaceae	*Cynodon plectostachyus*	38	3.3
Poaceae	*Sacciolepis africana*	65	5.7
Poaceae	*Cymbopogon commutatus*	35	3.1

Poaceae	*Hyparrhenia glabriuscula*	30	2.6
Poaceae	*Cynodon nlemfuensis*	43	3.8
Poaceae	*Cymbopogon citratus*	37	3.3
Poaceae	*Hyparrhenia rufa*	19	1.7
Poaceae	*Hyparrhenia hirta*	27	2.4
Poaceae	*Hyparrhenia cymbaria*	7	0.6
Poaceae	*Panicum maximum*	5	0.4
Poaceae	*Oryza sativa*	3	0.3
Poaceae	*Echinochloa cruspavonis*	68	6
Poaceae	*Leptochloa rupestris*	56	4.9
Cyperaceae	*Cyperus rigidifolius*	83	7.3
Fabaceae	*Vigna vexillata*	5	0.4
Fabaceae	*Chamaeurista mimosoides*	3	0.3
Fabaceae	*Psoralea bituminosa*	2	0.2
Fabaceae	*Laburum anagyroide*	6	0.5
Onagraceae	*Epilobium hirsutum*	8	0.7
Commelinaceae	*Commelina benghalensis*	35	3.1
Amaranthaceae	*Alternanthera nodiflora*	67	5.9
Asteraceae	*Guizotia scabra*	8	0.7
Asteraceae	*Galinsoga parviflora cov.*	5	0.4
Apiaceae	*Hydrocotyle mannii*	42	3.7
Combretaceae	*Combretum pancultum*	6	0.5
Lamiaceae	*Orthosiphon schimperi*	31	2.7
Polygonaceae	*Fallopia baldschanira*	15	1.3
Polygonaceae	*Persicaria decipiens*	17	1.5
Polygonaceae	*Persicaria attenuate*	24	2.1
Typhaceae	*Typha latifolia*	107	9.4
	Total	1138	100

Table 4: Plant species consumed by hippos during the wet and dry seasons in DWS

**Species**	**Wet season**	**Dry season**	**Total**
Echinochloa pyramidalis	20 (2.3%)	40 (15.1%)	62 (5.4 %)
Coelorhachis afraurita	4 (0.5%)	2 (0.8%)	6 (0.5%
Eriochloa fatmensis	80 (9.2%)	19 (7.2%)	99 (8.7%)
Cynodon dacylon	60 (6.9%)	15 (5.7%)	75 (6.6%)
Cynodon plectostachyus	28 (3.2%)	10 (3.8%)	38 (3.3%)
Sacciolepis africana	56 (6.4%)	9 (3.4%)	65 (5.7%)
Cymbopogon commutatus	22 (2.5%)	13 (4.9%)	35 (3.1%)

Hyparrhenia glabriuscula	30 (3.4%)	-	30 (2.6%)
Cynodon nlemfuensis	23 (2.6%)	20 (7.5%)	43 (3.8%)
Cymbopogon citratus	21 (2.4%)	17 (6.4%)	37 (3.3%)
Hyparrhenia rufa	14(1.6%)	5 (1.9%)	19 (1.7%)
Hyparrhenia hirta	20 (2.3%)	7 (2.6%)	27 (2.4%)
Hyparrhenia cymbaria	5 (0.6%)	2 (0.8%)	7 (0.6%)
Panicum maximum	5 (0.6%)	-	5 (0.4%)
Oryza sativa	3 (0.3%)	-	3 (0.3%)
Echinochloa cruspavonis	50 (5.7%)	18 (6.8%)	68 (6%)
Leptochloa rupestris	45 (5.2%)	11 (4.2%)	56 (4.9%)
Cyperus rigidifolius	65 (7.4%)	18 (6.8%)	83 (7.3%)
Vigna vexillata	3 (0.3%)	2 (0.8%)	5 (0.4%)
Chamaeurista mimosoides	2 (0.2%)	1 (0.4%)	3 (0.3%)
Psoralea bituminosa	2 (0.2%)	-	2 (0.2%)
Laburum anagyroide	6 (0.7%)	-	6 (0.5%)
Epilobium hirsutum	-	8 (3%)	8(0.7%)
Commelina benghalensis	30 (3.4%)	5 (1.9%)	35 (3.1%)
Alternanthera nodiflora	67 (7.7%)	-	67 (5.9%)
Guizotia scabra	8 (0.9%)	-	8 (0.7%)
Galinsoga parviflora cov.	5 (0.6%)	-	5 (0.4%)
Hydrocotyle mannii	30 (3.4%)	12 (4.5%)	42 (3.7%)
Combretum pancultum	-	6 (2.3%)	6 (0.5%)
Orthosiphon schimperi	31 (3.6%)	-	31 (2.7%)
Fallopia baldschanira	11 (1.3%)	4 (1.5%)	15 (1.3%)
Persicaria decipiens	17 (1.9%)	-	17 (1.5%)
Persicaria attenuate	24(2.7%)	-	24 (2.1%)
Typha latifolia	86 (9.9%)	21 (7.9%)	107 (9.4%)
Total	873 (76.7%)	265 (23.3%)	1138 (100%)

Table 5: Mean percentage of food items identified from faecal analysis of hippopotamus in the study area

	Wet season				Dry season			
Samples	Graminoids	Herbs	Dicots	Unknown	Graminoids	Herbs	Dicots	Unknown
1	50	30	10	10	50	20	20	10
2	60	20	20	-	80	20	-	-
3	40	50	10	-	50	20	20	10

4	30	40	10	20	90	0	10	-
5	50	30	-	20	60	0	30	10
6	70	20	-	10	70	20	-	10
7	70	30	-	-	80	0	20	-
8	40	40	20	-	50	30	-	20
9	60	-	30	10	60	10	10	20
10	50	30	20	-	80	10	10	-
11	60	30	-	10	90	0	-	10
12	40	50	10	-	70	20	10	-
13	70	30	-	-	30	30	30	10
14	80	20	-	-	70	30	-	-
15	50	30	20	-	40	30	20	10
16	80	20	-	-	60	0	30	10
17	60	-	30	10	50	30	20	-
18	50	40	-	10	60	40	-	-
19	30	60	10	-	70	0	20	10
20	50	20	20	10	80	20	-	-
Mean	54.5	29.5	10.5	5.5	64.5	16.5	12.5	6.5

Figure Click here to access/download;Figure;Fig 1.tif,Fig2.eps,etc..docx

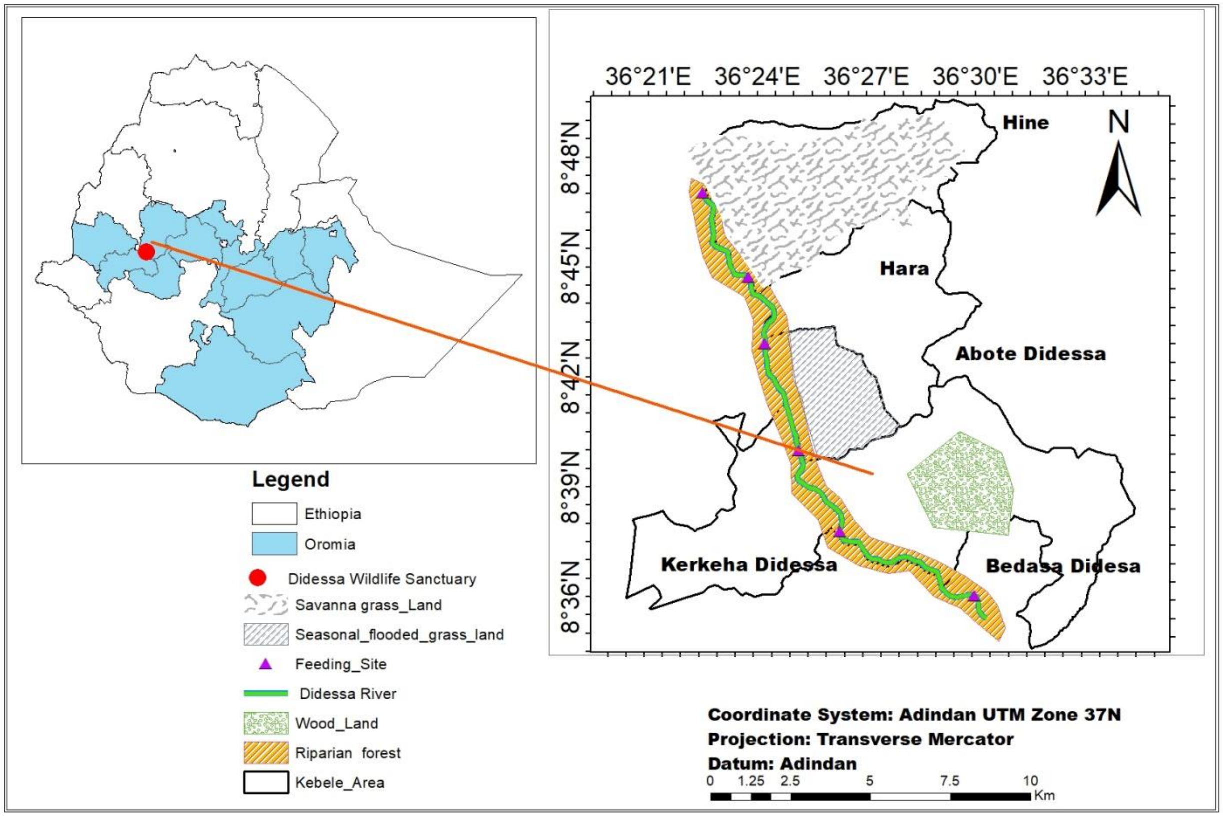

Figure 1: Map of study area (our team used shape files provided by the Ethiopian Mapping Agency to produce the map. The agency's official website offers free access to these shape files (http://www.ema.gov.et).

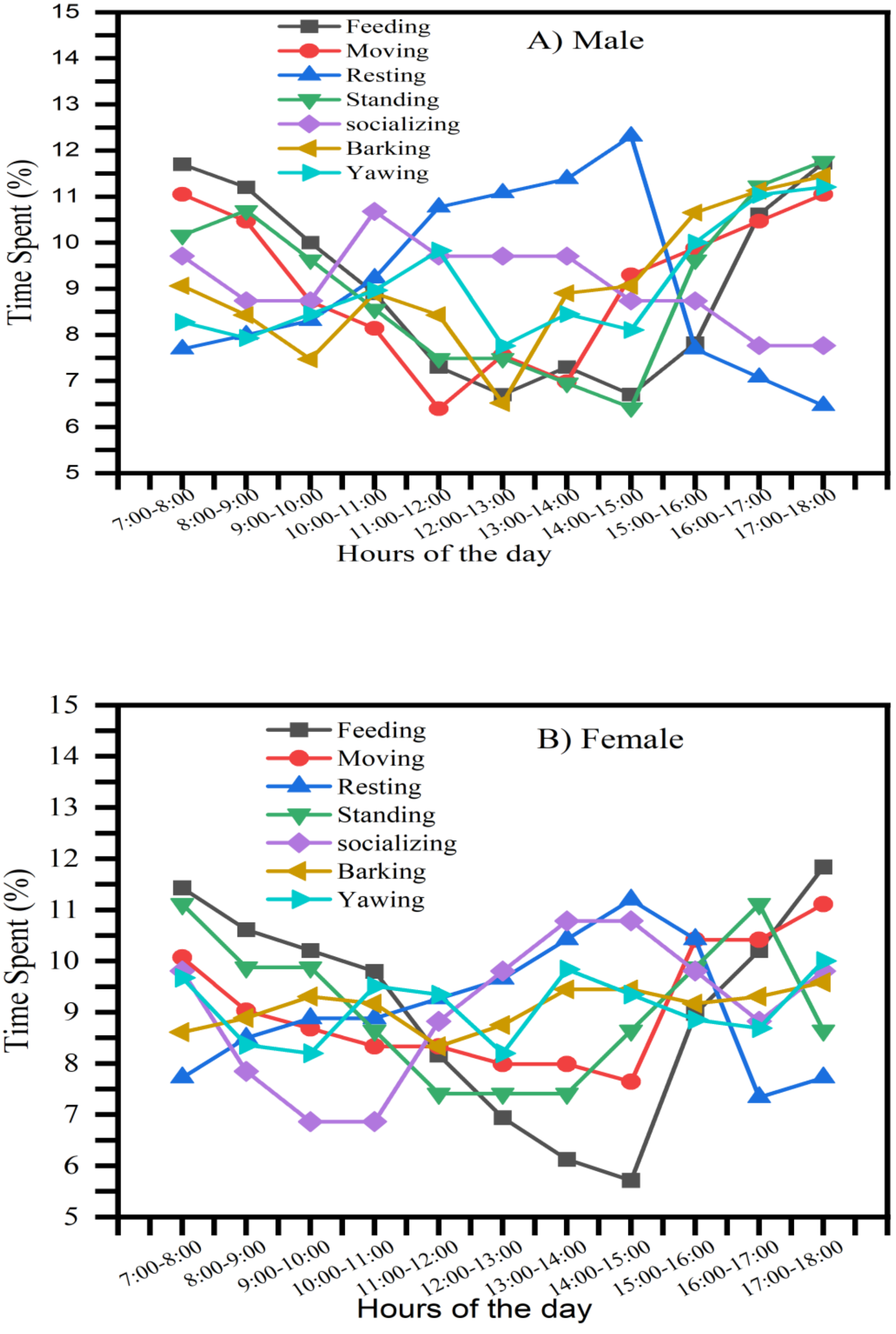

Figure: 2: The percentages of time specific activity budgets at different time of the day in the study area

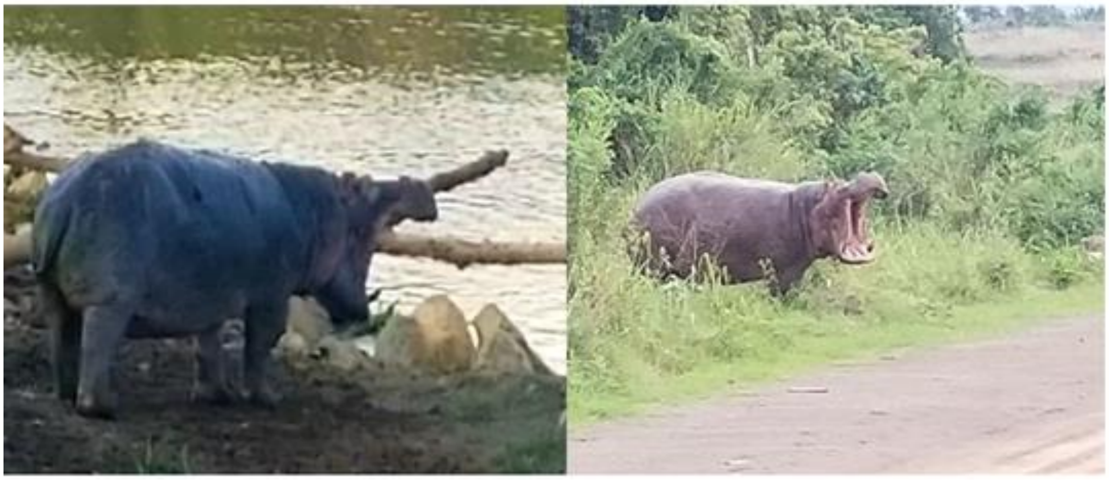

Figure 3: Yawning characteristics of hippopotamus in the study area

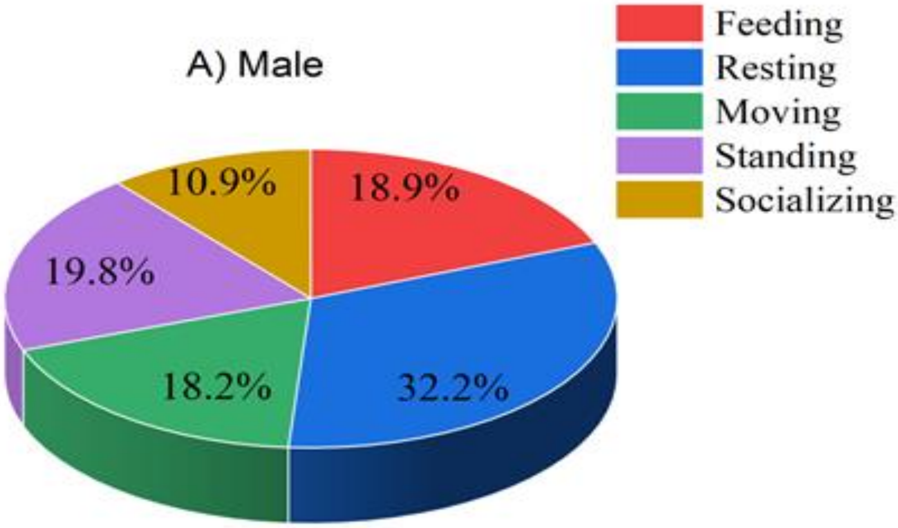

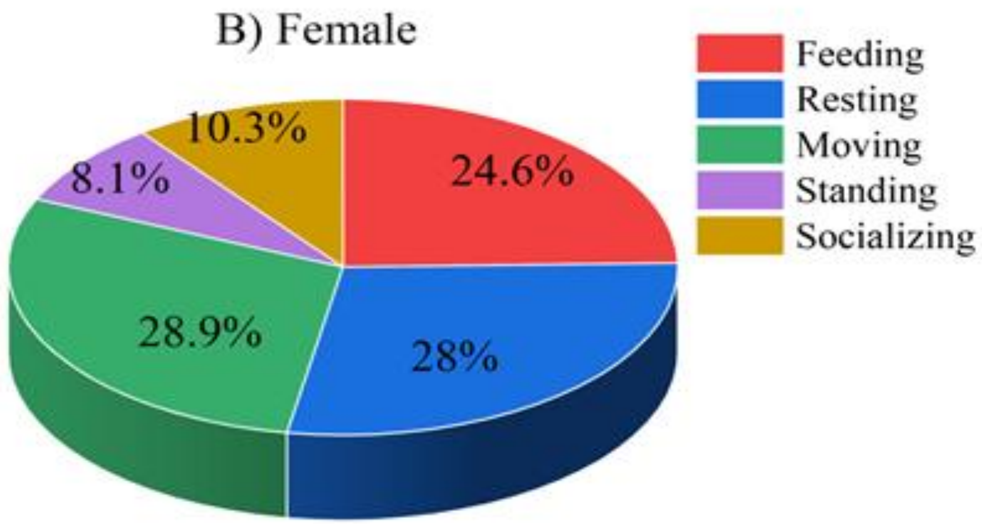

Figure 4: Major behavioural activities by male and female hippopotamus

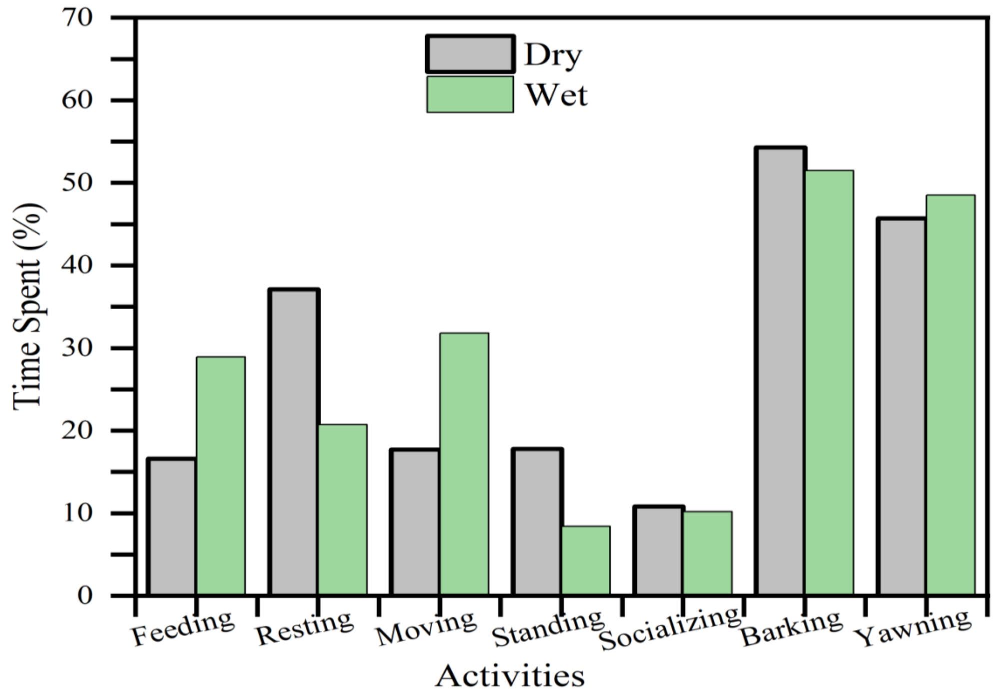

Figure 5: Activities of hippopotamus between wet and dry seasons in the study area

Click here to access/download
Supporting Information
Supplamentry.docx
