# Supplementary material for: Feeding ecology and activity patterns of Hippopotamus amphibious in the Dhidhessa Wildlife Sanctuary, Southwestern Ethiopia
Source: PLoS One. 2025 Aug 8;20(8):e0325731. doi: 10.1371/journal.pone.0325731 (PMC12334000; doi:10.1371/journal.pone.0325731)
Supplement: Attachment — Submitted filename: renamed_7d803.docx [file pone.0325731.s003.docx]

**Date; - March, 28, 2025**

**To: Munir Ahmad, PhD.**

**Academic Editor**

**Journal of PLOS ONE**

**Subject: Responses to the reviewers’ comments**

**Dear Dr. Stephanie S. Romanach,**

We are very grateful for your and reviewers’ critical and constructive comments regarding our manuscript numbered **[PONE-D-24-55622] - [EMID: 8a6d12479cab2714]**, entitled *“Feeding ecology and activity patterns of Hippopotamus amphibious in the Dhidhessa Wildlife Sanctuary, Southwestern Ethiopia”.* Please find re-submitted (the revised version) of our manuscript in which we have carefully considered and addressed all comments raised by the referees. The revised version of the manuscript includes many improvements and summarized below. Moreover, the revised manuscript is presented using track-change with red color. We hope our responses are satisfactory and you will find this version suitable for publication in Journal of PLOS ONE. We look forward to hearing from you.

Dear Academic Editor, please note the following points

1. Our responses to the reviewers’ comments are provided
2. The line numbers with red colors (*line#)* refer to the improved version of the revised manuscript

Best regards,

Corresponding author

**Response to Academic editor #1**

1. **Comment 1(General comment),** the reviewers suggested that the manuscript when submitting your revision, we need you to address these additional requirements.
- The manuscript should meet PLOS One's style requirements.
- Data availability by the authors upon reasonable request.
- Figures 1 in your submission contain [map/satellite] images, which may be copyrighted.

***Response*:** Thank you very much for your suggestion. We agree with the comment and we made improvements to the issues mentioned above.

2. **Comment 2:** Methods and choice of two focal individuals to generalize the behavior are of major concern of common hippos in the DWS. For this, statistical methods need to be clear.

***Response*:** Thank you very much for your suggestion. We agree with the comment and made correction from Line# 126-196. We have re-written or revised the whole of portions the methods.

3. **Comment 3:** Sweeping conclusions on the community aspects, which are not part of the study, may be removed.

***Response*:** Thank you very much for your comments and suggestions. We have made correction as commented on *Line#* 402-426.

4. **Comment 4:** “Classification of behaviors within the context of the study and whether the statistical analysis of the feeding ecology included the faecal samples or only the feeding observations, needs clarification “

***Response*:** Thank you very much for your suggestions. We have made correction as commented on *Line#* 161-196.

5. **Comment 5:** Certain parts of the manuscript need to be rewritten to address grammar and syntax where the text is difficult to understand

***Response*:** Thank you very much for your comments and suggestions. We have changed and revised as commented.

6. **Comment 6:** The reviewers suggested that Please clarify whether written consent for publication has been obtained from the participants shown in [FIGURE S1] in the ‘Consent for publication’ section. The manuscript when submitting your revision, we need you to address these additional requirements.

**Response:** Thank you very much for your suggestion. Microscopic imaging for fecal analysis was conducted by the corresponding author. Informed consent for publication has been duly obtained from all participants involved in the study.

7. **Comment 7:** When completing the data availability statement of the submission form, you indicated that you will make your data available on acceptance.

**Response:** Thank you very much for your comments. We agree with the comment and made correction based as commented.

8. **Comment 8:** We note that you have referenced Timbuka, C. D. which has currently not yet been accepted for publication. Please remove this from your References and amend this to state in the body of your manuscript: (Timbuka, C. D

***Response*:** Thank you very much for your suggestions. We agree with the comment and made correction by rewriting the words using a track change from Line# 497

9. **Comment 9, Line 69:** Unclear on what ‘dimensions’ means here, consider rephrasing for clarity

**Response:** Thank you very much for your suggestions. We have made correction on line #71 – 73. For clarity the dimensions can include things like temperature, food availability, pH, salinity, and other factors that influence an organism's survival and reproduction.

10. **Comment 10, Line 84:** What were the ‘intended purposes’ of the formation of DWS? Also, suggest providing the full name here of DWS as done in abstract.

**Responses:** Thank you very much for your comments. We agree with the comment and made correction. Based on the comments and suggestions using a track change from Line# 37,96-100, we have re-written or revised the whole of those portions.

11. **Comment 11**, **98:** remove ‘were’

***Response*:** Thank you very much for your comments/suggestions. We agree with the comment and made correction (line #108).

12. **Comment 12, Line 115:** ‘primarily growing’ as in Hyparrhenia spp. are dominant? Or perennially? Maybe clarify

***Response*:** Thank you very much for your suggestions. We agree with the comment and made correction *from Line#* 122-124.

13. **Comment 13,** **line 127-132:**   “the addition of ‘actions’ and ‘occurrences’ is confusing, stick to ‘activities’ and ‘events’ as per the analysis?

***Response*:** Thank you very much for your comments. We agree with the comment and made correction *from Line#* 147-150.

14. **Comment 14,** **Line 131-132:** State why this distinction was made and what was considered a short period of time vs a long period of time

***Response*:** Thank you very much for your comments and suggestions. We agree with the comment and made correction using a track change *from* *Line#* 126-160. ‘’Behavioral activities were defined as sustained actions of relatively longer duration, including: Resting, Standing, Walking, Feeding, Socializing. Behavioral events comprised discrete, short-duration patterns, often lasting seconds, such as: Vocalizations (e.g., barking) Transient body movements (e.g., yawning) “

15. **Comment 15, Line 129+177-178:** what types of behaviors were placed in the ‘socialisation’ category? Also specify the behaviors considered ‘mating behaviors’ and how these differ from yawning and barking (as mentioned in the discussion on line 296)

***Response*:** Thank you very much for your comments. We agree with the comment and made correction using a track change *from* *Line#* 126-160.

16. **Comment 16,** **Line 198-199:** What about behavioural events as per your definitions?

***Response*:** Thank you very much for your comments and suggestions. We agree with the comment and made correction using a track change *from* *Line#* 141-160

17. **Comment 17, Line 218-219:** suggest rephrasing ‘feeding bites’ for clarity as unclear what this means

***Response*:** Thank you very much for your suggestions. We agree with the comment and clarified. ‘’To clarify the term "feeding bites," alternative phrasings can be used depending on the focus of observation: "Bites during foraging" explicitly links the action to feeding temporality, while "plant material bites" specifies the target (e.g., grasses, aquatic vegetation) if describing grazing specificity’’.

18. **Comment 18,** **Line 239:** Suggest remove ‘importantly’.

***Response*:** Thank you very much for your comments and suggestions. We agree with the comment and made correction using a track change *from* *Line#* 276-277.

19. **Comment 19,** **Line 238-242:**   Does this statistical test include the faecal samples between the wet and dry seasons as well as the observations? If not, the manuscript would benefit from an additional statistical test of the faecal samples to determine whether the observed differences between the seasons were statistically significant.
    ***Response*:** Thank you very much for your comments. However, we made the comparisons based on the methodologies and objectives rather than the area's size *from* *Line#* 285-293.
20. **Comment 20,** **Line 256-279:** Repeated paragraph, suggest remove the second one as lines 277-279 are incomprehensible.

***Response*:** Thank you very much for your comment. We agree with the comment and made correction using a track change from Line# 327-345

21. **Comment 21,** **Line 283:** Add references to support these statements, no analysis in this study was undertaken to conclude that these variables significantly altered the activity patterns.

***Response:*** Thank you very much for your comment. We agree with the comment and made correction using a track change *from* *Line#* 322.

22. **Comment 22,** **Line 332-336:** Suggest rephrasing ‘almost all’ since the removal of 12 species out of a total of 34 is a significant amount.

***Response*:** Thank you very much for your comments and suggestions. We agree with the comment and made correction using a track change *from* *Line#* 366-373.

23. **Comment 23,** **Line 345-346:** Given the consistency of the results with earlier studies in Ethiopia, how could the outcome of the research contribute to the management of other areas with hippo populations

***Response*:** Thank you very much for your suggestions. We have changed as commented, on #Line 198-293.
